# Counseling and Cardiovascular Disease Risk Factor Control in Long-Term Cancer Survivors: A Randomized Clinical Trial

**DOI:** 10.1001/jamanetworkopen.2025.55863

**Published:** 2026-02-05

**Authors:** Eric J. Chow, Yan Chen, Yutaka Yasui, Laura-Mae Baldwin, Melissa M. Hudson, Tammy M. Muller, Paul C. Nathan, Siu L. Ngai, Timothy J. D. Ohlsen, Claire Snyder, Karen L. Syrjala, Emily S. Tonorezos, Gregory T. Armstrong, Kevin C. Oeffinger

**Affiliations:** 1 Fred Hutchinson Cancer Center, Seattle, Washington; 2 University of Washington, Seattle; 3 St Jude Children’s Research Hospital, Memphis, Tennessee; 4 Hospital for Sick Children, University of Toronto, Toronto, Ontario, Canada; 5 Johns Hopkins School of Medicine, Baltimore, Maryland; 6 Weill Cornell Medicine, New York, New York; 7 Duke University, Durham, North Carolina

## Abstract

**Importance:**

Survivors of childhood cancer have an increased risk of cardiovascular disease (CVD). However, many survivors at high risk do not receive recommended CVD screening tests as young adults.

**Objective:**

To assess whether a survivorship care plan (SCP)–based counseling intervention improves CVD risk factor control in high-risk survivors.

**Design, Setting, and Participants:**

The Communicating Health Information and Improving Coordination With Primary Care (CHIIP) Study was a randomized clinical trial that enrolled eligible participants from the Childhood Cancer Survivor Study cohort in 9 US metropolitan areas between August 2017 and April 2020, with follow-up completed July 2022. Participants included adult survivors of childhood cancer exposed to cardiotoxic cancer therapies with undertreated CVD risk factors (hypertension, dyslipidemia, and glucose intolerance defined by standard guidelines); of 1840 survivors approached, 842 consented to participate, and 347 met all eligibility requirements and were randomized after a baseline home assessment. Data analysis was completed March 18, 2025.

**Intervention:**

The intervention consisted of a single remote session to review measurements and a SCP with personalized CVD risk information and to develop a CVD risk factor management plan, with a booster session 4 months later. Enhanced care controls received measurements with abnormal findings noted and written encouragement to follow up with their primary care clinician (PCC). PCCs received all materials sent to participants.

**Main Outcomes and Measures:**

Blood pressure, lipid profile, and glucose and hemoglobin A_1c_ levels collected by a trained home examiner at baseline and 1-year follow-up, with undertreatment defined by standard guidelines.

**Results:**

A total of 347 cancer survivors (mean [SD] age, 40.5 [9.4] years; 182 [52.4%] male) were randomized, 175 to the intervention group and 172 to the enhanced care control group. Of these, 194 participants (53.0%) participants had undertreated hypertension at baseline; 180 (51.9%), undertreated dyslipidemia; and 170 (49.0%) undertreated glucose intolerance. After 1 year, 45 of 173 surviving intervention participants (26.0%) and 52 of 172 enhanced care control participants (30.2%) had less undertreatment, with lower percentages for each condition vs baseline. Although the intervention did not reduce undertreatment compared with the control condition (odds ratio, 1.31; 95% CI, 0.84-2.05), greater engagement was associated with less undertreatment at 1 year among intervention participants (odds ratio, 0.31; 95% CI, 0.18-0.72). The intervention group had improved PCC documentation of CVD risk vs controls after 1 year (14.8% improvement vs 0.9%; *P* = .002).

**Conclusions and Relevance:**

In this randomized clinical trial of long-term survivors of childhood cancer, the addition of survivorship-based self-management counseling did not reduce undertreatment beyond simply providing CVD risk assessments to survivors and PCCs. Additional strategies to mitigate CVD risk in high-risk survivors should be examined in the future.

**Trial Registration:**

ClinicalTrials.gov Identifier: NCT03104543

## Key Points

**Question:**

Among adult survivors of childhood cancer with undertreated hypertension, dyslipidemia, or glucose intolerance, can remotely delivered, clinician-led, self-management counseling sessions reduce undertreatment more than provision of screening results alone?

**Findings:**

In a randomized clinical trial of 347 participants, there was no significant difference after 1 year between the study groups: 26% of intervention participants and 30% of enhanced usual care control participants had less undertreatment of cardiovascular disease risk factors.

**Meaning:**

In this study, self-management counseling did not reduce cardiovascular risk factor undertreatment beyond simply providing hypertension, lipid level, and diabetes screening results to high-risk cancer survivors and their primary care clinicians.

## Teaser

This randomized clinical trial assesses whether a survivorship care plan with remotely delivered counseling increases control of risk factors for cardiovascular disease among adult survivors of childhood cancer.

## Introduction

Cardiovascular disease (CVD) is a leading contributor to morbidity and mortality in cancer survivors.^1^ This includes childhood cancer survivors who now have 5-year survival rates of 85% or greater^2^ but experience a substantial burden of CVD and other chronic illness during subsequent decades. Among those exposed to cardiotoxic treatments (ie, anthracyclines, chest radiotherapy), the cumulative incidences of ischemic heart disease and heart failure can exceed 10% by 50 years of age.^3,4^ With an estimated half million survivors of childhood cancers living in the US, this is an increasingly important public health problem.^5^

Multiple studies in survivors of adult and childhood cancers have shown that even after considering cancer treatments, the presence of conventional CVD risk factors such as hypertension, dyslipidemia, and glucose intolerance further increase risk of serious CVD in a more than additive fashion.^1,6^ Among childhood cancer survivors, the development of these conditions occurs at much younger ages compared with the general population.^7^ Prior research has also shown that more than 80% of adult survivors of childhood cancer are followed up by primary care clinicians (PCCs).^8,9,10^ Given that these conditions typically present in older adults and the limited knowledge of cancer survivor–specific screening guidelines among PCCs,^11,12^ most younger adult-aged survivors at high risk do not receive recommended CVD screening tests.^13,14^

To determine whether it is possible to improve control of conventional CVD risk factors among adult-aged survivors of childhood cancer, we conducted a randomized clinical trial that enrolled survivors estimated to be at high risk of early ischemic heart disease or heart failure due to prior anthracycline and/or chest radiotherapy exposure and who were found to have undertreated hypertension, dyslipidemia, and glucose intolerance. The study’s intervention featured a National Academy of Medicine–recommended survivorship care plan (SCP) coupled with 2 brief, remotely delivered counseling sessions with survivorship-trained advanced practice professionals (APPs), approximating typical clinical encounters. SCPs are designed to promote knowledge and awareness of personal health risks among survivors and to disseminate that information to PCCs.^15^ After 1 year, the study assessed participants’ blood pressures, lipid profiles, and glucose tolerance to determine the intervention’s efficacy compared with controls. Results from this study can inform the evidence base for optimal delivery of health information and care coordination for high-risk cancer survivors and their PCCs.

## Methods

### Study Population

Details of the Communicating Health Information and Improving Coordination With Primary Care (CHIIP) Study have been published.^14,16,17^ The trial protocol is provided in Supplement 1, and the study followed the Consolidated Standards of Reporting Trials (CONSORT) reporting guideline. Participants were recruited from the Childhood Cancer Survivor Study (CCSS), a cohort of survivors of common childhood cancers diagnosed between 1970 and 1999 (survival ≥5 years).^18^ CHIIP-specific eligibility criteria included: (1) 18 years or older at consent; (2) estimated increased risk of future ischemic heart disease or or heart failure (ie, ≥10% risk by 50 years of age)^3,19^; (3) living within 50 miles of 9 US metropolitan areas (eTable 1 in Supplement 2); and (4) ability to speak English. Participants with ischemic heart disease or heart failure and those receiving active cancer treatment were excluded. Recruitment began August 2017 and stopped April 2020 due to the COVID-19 pandemic. The study was approved by review boards at the Fred Hutchinson Cancer Center and St. Jude Children’s Research Hospital. Participants had the option of providing written, verbal, or electronic informed consent.

### Baseline Assessment

Participants completed a survey assessing medical history, lifestyle, health-related self-efficacy,^20^ and the Multidimensional Health Locus of Control (MHLC) scale.^21^ Participants then underwent a standardized home assessment with height, weight, waist circumference, resting blood pressure (3 seated measurements ≥3 minutes apart), and a venous blood draw for a lipid profile and glucose and hemoglobin A_1c_ (HbA_1c_) levels. Participants were not required to fast, but the duration of fasting was recorded.^22^ Samples were shipped and processed by a Clinical Laboratory Improvement Amendments (CLIA)–certified central laboratory. Because of COVID-19, baseline assessments after March 2020 were performed at community-based CLIA-certified facilities (ie, Labcorp, Quest Diagnostics Inc).

Irrespective of current treatment status, participants who had any of the following abnormal findings corresponding to potentially undertreated hypertension,^23^ dyslipidemia,^24,25,26^ or glucose intolerance^27^ were eligible for randomization: (1) mean of the lowest 2 blood pressure measurements 130/80 mm Hg or greater; (2) low-density lipoprotein (LDL) cholesterol level 160 mg/dL or greater (to convert to mmol/L, multiply by 0.0259); (3) triglyceride level 150 mg/dL or greater after fasting at least 10 hours or 200 mg/dL or more after fasting less than 10 hours (to convert to mmol/L, multiply by 0.0113); (4) glucose level 100 mg/dL or greater after fasting 8 hours or longer without known diabetes (to convert to mmol/L, multiply by 0.0555) or 140 mg/dL or greater after fasting less than 8 hours without known diabetes; or (5) HbA_1c_ level 5.7% or greater for those not known to have diabetes or 7.0% or greater for those known to have diabetes. The threshold of 130/80 mm Hg for blood pressure was chosen as it corresponds to stage 1+ hypertension,^23^ and hypertension, dyslipidemia, and glucose intolerance are all recommended targets for intervention for patients at increased risk of ischemic heart disease and heart failure without structural heart disease and/or symptoms (stage A).^28,29^

### Randomization and Intervention Assignment

Participants were randomized 1:1, stratified by site, to intervention vs control. Intervention participants received a manualized, remotely delivered, clinician-led (by a survivorship-trained APP), SCP-focused self-management session approximating a standard clinical encounter. Participants were mailed an SCP (eAppendix in Supplement 2) with their personalized cancer treatment history, recommendations, and results from their baseline assessment. The SCP provided absolute and relative risk estimates of ischemic heart disease or heart failure compared with the general population, based on each participant’s prior cancer treatment.^3,19^ Participants were scheduled a 30-minute phone or video session with the study clinician to review the SCP and create an action plan with mutually agreed-upon goals to address underlying CVD risk factors, based on chronic disease self-management models.^30,31,32^ Afterward, the personalized action plan was mailed to the participant. Four months after the initial session, participants scheduled a 15-minute phone or video follow-up session with the study clinician to review the plan, address barriers, and mutually agree on a revised plan if needed. The clinician then rated the participant’s engagement with and completion of the action plan.

Participants assigned to enhanced usual care received a copy of their assessment results, with a recommendation to seek medical follow-up for abnormal values. The rationale for this control condition was that although many of these younger adult participants likely would not have received these screening tests as part of routine care per general population guidelines, it was deemed unethical to withhold potentially actionable results from them. At 4 months, controls were mailed a generic letter of thanks with a reminder of their 1-year follow-up.

### Follow-Up Assessment

After 1 year, participants completed an abridged survey and a second home visit where repeat anthropometric measurements and blood samples were obtained. For participants assessed after March 2020, the study mailed scales and blood pressure cuffs to participants plus a kit to self-collect capillary blood using dried blood spot (DBS) methodology. Participants were instructed to self-measure their weight and resting blood pressure (3 times); DBS were centrally assayed for lipid profile and levels of glucose and HbA_1c_.^33,34^ As DBS assays were not CLIA certified, those results were not released to participants or their PCCs. For both study groups, PCCs were concurrently mailed a copy of all materials sent to participants from all time points. All follow-up was completed as of July 2022.

### Medical Records

Ambulatory records were obtained from participants’ PCCs starting 2 years prior to trial enrollment through the 1-year follow-up.^14^ Records were evaluated with regard to documentation of cancer history, cardiotoxic treatment exposures, language noting that participants were considered at greater CVD risk, the presence of any SCP or late-effects surveillance plan, and whether more cardiac testing (eg, electrocardiography, echocardiography, or other imaging) was performed or planned.

### Covariates

Prespecified covariates included sex, age at 1-year follow-up, time since cancer diagnosis, and baseline body mass index (≥30 vs <30; calculated as weight in kilograms divided by height in meters squared), health insurance status, and recent (≤2 years) visit to a survivorship clinic. Self-reported race and ethnicity data were collected for this National Institutes of Health–funded study due to requirements in accrual and annual reporting. Categories included Hispanic, non-Hispanic Asian or Pacific Islander, non-Hispanic Black, non-Hispanic White, and other (including American Indian or Alaska Native or other) or unknown race or ethnicity.

### Statistical Analysis

The prespecified statistical analysis plan sought to determine whether the intervention assignment was associated with a lower likelihood of having an undertreated CVD risk factor (ie, hypertension, dyslipidemia, and/or glucose intolerance still meeting the thresholds defined at baseline) compared with the control condition at 1-year follow-up. As participants may have contributed as many as 3 outcomes, we used a logistic regression model with a generalized estimating equation modification to estimate the overall intervention effect as a single parameter (odds ratio [OR]) and account for the 3 outcomes’ intraindividual correlation.^35^ Analyses were conducted per intention to treat and included all randomized participants except those who died before follow-up (n = 2). Participants who lacked 1-year follow-up were assumed to have the same values as at baseline. Due to the nature of the intervention, participants could not be blinded. However, home examiners, medical record abstractors, and statisticians (Y.C. and Y.Y.) were blinded to randomization assignment.

Other preplanned analyses explored whether participant engagement and action plan completion at 4 months influenced intervention efficacy, and whether the intervention influenced PCC practices as documented by medical records. We also examined the association of self-efficacy and MHLC domains with risk of undertreatment at 1 year.^17,36^ Changes in individual parameters (blood pressures and levels of LDL cholesterol, triglyceride, glucose, and HbA_1c_) were assessed via linear regression adjusting for each parameter’s baseline value.

The trial was designed with 800 participants having baseline assessments, 480 (60.0%) meeting eligibility criteria and randomized, and 380 having 1-year follow-up (20% attrition), allowing the study to have 80% power to detect a 12% difference between the study groups if the control group experienced a 10% improvement. If the control group had a 50% improvement, the study would be powered to detect a 29% difference. Analyses were conducted using SAS, version 9.4 (SAS Institute Inc). Reported *P* values were all 2 sided, with *P* < .05 considered statistically significant. Primary analyses were completed March 18, 2025.

## Results

**Figure 1.**
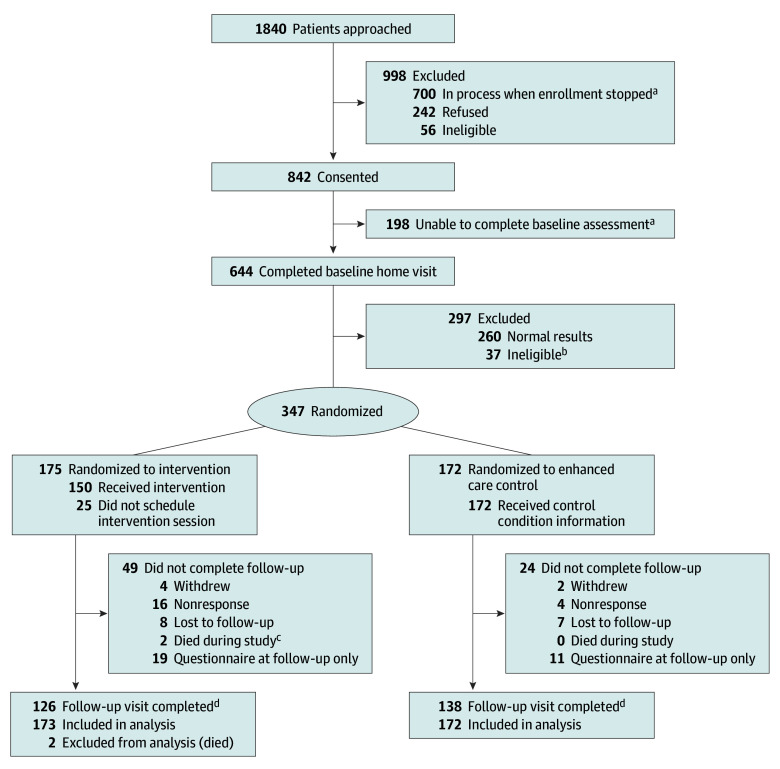
Study Flow Diagram ^a^Active recruitment and screening of participants and in-home assessments stopped as of April 2020 due to the COVID-19 pandemic. ^b^Includes those found on baseline questionnaire to be ineligible despite passing initial consent screener (n = 30), refused upfront randomization (n = 6), and blood sample lost and unable to recollect (n = 1). ^c^Includes deaths unrelated to study participation. ^d^Due to COVID-19 pandemic and inability to continue in-home assessments, 58 intervention and 60 control participants had follow-up assessments completed via dried blood spots and self-measured blood pressures.

Among 1840 individuals approached, 842 consented to participate and 644 (76.5% of consented) completed a baseline assessment (Figure 1). Among 700 participants whose participation status was not yet finalized as of April 2020, more than 400 had not exhausted recruitment approaches. Overall, 347 participants met eligibility for the trial (165 [47.6%] female and 182 [52.4%] male; mean [SD] age, 40.5 [9.4] years; and mean [SD] time since cancer diagnosis, 31.1 [7.8] years) with 264 (76.1%) completing 1-year follow-up (118 via DBS and self-measured blood pressures). In terms of race and ethnicity, 30 participants (8.6%) were Hispanic; 8 (2.3%), non-Hispanic Asian or other Pacific Islander; 13 (3.7%), non-Hispanic Black; 293 (84.4%) non-Hispanic White; and 3 (0.9%), other or unknown race or ethnicity. In general, characteristics of the randomized groups (175 intervention and 172 enhanced care control) were similar, with 310 (89.3%; 155 in each group) having had a routine checkup in the past 2 years (Table 1).

**Table 1.** Baseline Characteristics of 347 Participants

Characteristic	Patient group	
	Intervention (n = 175)	Enhanced care control (n = 172)
Age at cancer diagnosis, mean (SD), y	9.5 (5.6)	9.3 (5.7)
Age at start of study, mean (SD), y	41.4 (9.9)	39.6 (8.8)
Sex, No. (%)		
Female	86 (49.1)	79 (45.9)
Male	89 (50.9)	93 (54.1)
Race and ethnicity, No. (%)		
Hispanic	17 (9.7)	13 (7.6)
Non-Hispanic Asian or Pacific Islander	5 (2.9)	3 (1.7)
Non-Hispanic Black	5 (2.9)	8 (4.7)
Non-Hispanic White	146 (83.4)	147 (85.5)
Other or unknown^a^	2 (1.1)	1 (0.6)
Cancer type, No. (%)		
Leukemia	65 (37.1)	48 (27.9)
Central nervous system tumor	19 (10.9)	14 (8.1)
Lymphoma	41 (23.4)	55 (32.0)
Bone or soft tissue sarcoma	27 (15.4)	26 (15.1)
Embryonal tumor (neuroblastoma, Wilms)	23 (13.1)	29 (16.9)
Anthracycline exposure, No. (%)	130 (74.3)	131 (76.2)
Chest radiation exposure, No. (%)	84 (48.0)	78 (45.3)
Current smoking or tobacco use, No. (%)	20 (11.4)	27 (15.7)
Health care utilization, No. (%)		
Health insurance coverage	160 (91.4)	161 (93.6)
Routine checkup past 1-2 y^b^	155 (88.6)	155 (90.1)
History of survivorship clinic attendance	66 (37.7)	71 (41.3)
Health-related self-efficacy *t* score, mean (SD)^c^	50.7 (11.5)	50.8 (11.0)
MHLC domain scores, mean (SD)^d^		
Internal	4.2 (0.8)	4.3 (0.8)
Chance	2.8 (0.8)	2.9 (0.8)
Powerful others	3.3 (0.9)	3.4 (0.8)
Physiologic measurements, median (IQR)		
BMI	28.1 (24.7-32.6)	27.2 (24.2-32.0)
Systolic blood pressure, mm Hg	119.0 (109.0-129.0)	118.0 (108.0-125.0)
Diastolic blood pressure, mm Hg	78.0 (69.0-84.0)	79.0 (70.0-83.0)
Total cholesterol level, mg/dL	191.0 (168.0-220.0)	193.0 (169.0-222.5)
HDL cholesterol level, mg/dL	44.0 (37.0-54.0)	46.0 (38.0-54.0)
LDL cholesterol level, mg/dL	111.7 (91.5-138.0)	108.3 (89.5-141.7)
Triglyceride level, mg/dL	168.5 (105.0-241.0)	157.5 (105.0-242.5)
Blood glucose level, mg/dL	89.0 (80.0-101.0)	93.0 (81.0-102.5)
HbA_1c_ level, %	5.6 (5.3-5.9)	5.6 (5.3-6.0)
Preexisting CVD risk factor undertreated, No. (%)^e^	92 (52.6)	74 (43.0)
Newly identified CVD risk factor, No. (%)^f^	121 (69.1)	138 (80.2)

Abbreviations: BMI, body mass index (calculated as weight in kilograms divided by height in meters squared); CVD, cardiovascular disease; HbA_1c_, hemoglobin A_1c_; HDL, high-density lipoprotein; LDL, low-density lipoprotein; MHLC, Multidimensional Health Locus of Control.

SI conversion factors: To convert cholesterol to mmol/L, multiply by 0.0259; glucose to mmol/L, multiply by 0.0555; triglycerides to mmol/L, multiply by 0.0113.

^a^ Includes American Indian or Alaska Native, other race or ethnicity, or unknown.

^b^ Defined as having had a routine checkup by a physician, physician assistant, or nurse practitioner.

^c^ Scores range from 12 to 70, with higher scores indicating greater self-efficacy.

^d^ Each domain score ranges from 1 to 6, with higher scores reflecting stronger support for that domain.

^e^ Includes known hypertension, dyslipidemia, or diabetes with values in the abnormal range.

^f^ Includes no known hypertension, dyslipidemia, or diabetes or prediabetes with values in the abnormal range.

**Figure 2.**
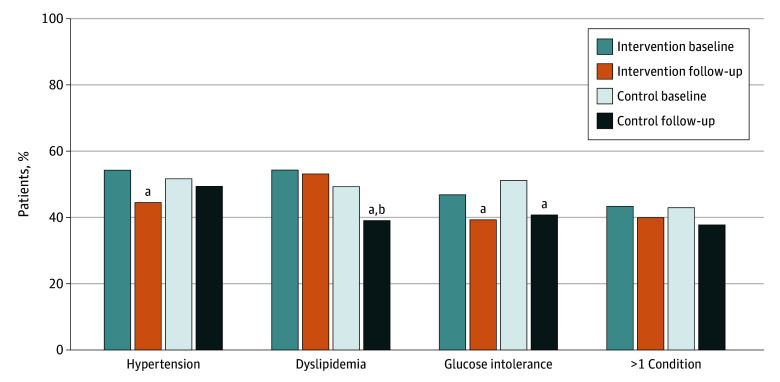
Percentage of Randomized Participants With Undertreated Target Conditions at Baseline and After 1-Year Follow-Up Target conditions include hypertension, dyslipidemia, and glucose intolerance. The intervention group included 175 participants at baseline and 173 at 1 year; the enhanced care control group, 172 at both time points. ^a^*P* < .05 for follow-up compared with baseline prevalence. All *P* values are provided in eTable 2 in Supplement 2. ^b^*P* = .009 for difference at follow-up between intervention compared with control groups, adjusting for baseline value.

At baseline, 184 participants (53.0%) had undertreated hypertension; 180 (51.9%), dyslipidemia; and 170 (49.0%), glucose intolerance. One hundred fifty participants (43.2%) had more than 1 undertreated condition, and 37 (10.7%) had all 3 (Figure 2 and eTable 2 in Supplement 2). After 1 year, rates of all 3 conditions, including rates of multiple conditions, were lower for both the intervention group and the control group. Overall, 45 of 173 surviving intervention participants (26.0%) and 52 of 172 control participants (30.2%) had less undertreatment after 1 year. If limited to those with 1-year follow-up (n = 264), 45 of 126 intervention participants (35.7%) and 52 of 138 control participants (37.7%) had less undertreatment. When differences in physiologic measurements were examined between groups, there were no significant differences (eTable 3 in Supplement 2). However, both groups had significantly lower diastolic blood pressure (−2.3 [95% CI, −4.0 to −0.5] and −1.9 [95% CI, −3.7 to −0.1] mm Hg) and LDL cholesterol (−18.0 [95% CI, −25.4 to −10.6) and −20.1 [95% CI, −28.9 to −11.2] mg/dL) values after 1 year compared with their respective baseline values. The intervention group also had significantly lower systolic blood pressures at follow-up vs baseline (−4.6 [95% CI, −7.1 to −2.1] mm Hg), while the control group had significantly lower triglyceride values at follow-up vs baseline (−48.9 [95% CI, −91.9 to −5.8] mg/dL).

When the overall risk of undertreatment was analyzed, the intervention did not reduce undertreatment after 1 year compared with the control (OR, 1.31; 95% CI, 0.86-2.00). The intervention effect was similar when the analysis was adjusted for covariates selected a priori (OR, 1.31; 95% CI, 0.84-2.05) (Table 2) and in sensitivity analyses if only participants who completed the 1-year follow-up were included (OR, 1.47; 95% CI, 0.94-2.29) or if outcomes were examined for those who completed the study by or after March 2020. However, in adjusted analyses, those who endorsed the MHLC domain *powerful others* were more likely to be undertreated (OR, 1.42; 95 CI, 1.08-1.87) (eTable 4 in Supplement 2), and those who had greater improvements in self-efficacy appeared to be less likely to be undertreated (OR for middle tertile, 0.56 [95% CI, 0.31-1.01]; OR for highest tertile, 0.64 [95% CI, 0.34-1.22]), although estimates were not statistically significant.

**Table 2.** Odds of Undertreatment After 1 Year Among Randomized Participants^a^

Characteristic	OR (95% CI)^b^
Study group	
Enhanced care control	1 [Reference]
Intervention	1.31 (0.84-2.05)
Sex	
Female	1 [Reference]
Male	1.41 (0.91-2.18)
Current age	1.01 (0.97-1.05)
Years after cancer diagnosis	1.00 (0.95-1.04)
Insurance coverage	
No	1 [Reference]
Yes	1.56 (0.67-3.67)
History of survivorship clinic attendance	
No	1 [Reference]
Yes	1.21 (0.78-1.88)
BMI	
<30	1 [Reference]
≥30	1.43 (0.90-2.27)
Undertreated condition	
Hypertension	1 [Reference]
Dyslipidemia	1.19 (0.75-1.87)
Diabetes	1.59 (1.00-2.52)

Abbreviations: BMI, body mass index (calculated as weight in kilograms divided by height in meters squared); OR, odds ratio.

^a^ Excludes 2 intervention participants who died before reaching the 1-year study time point (n = 345).

^b^ Adjusted for all variables shown.

Among 175 intervention participants, 150 (85.7%) completed baseline and 129 (73.7%) completed 4-month counseling sessions. Those rated as engaged (limited engagement, 29 [16.8%]; full engagement, 93 [53.8%]) with the action plan at 4 months were significantly less likely to be undertreated at 1 year vs those rated as unengaged (OR, 0.31; 95% CI, 0.13-0.72) (Table 3). Intervention participants who, regardless of engagement, had mostly (46 [26.6%]) or fully (42 [24.3%]) completed their action plan items at 4 months compared with those who had no plan completion were also less undertreated, although estimates were not statistically significant (OR, 0.54; 95% CI, 0.28-1.02).

**Table 3.** Association Between Levels of Action Plan Engagement and Completion Rates After 4 Months and Odds of Undertreatment After 1 Year Among Intervention Participants

Characteristic	No. (%) of participants (n = 173)	Risk of undertreatment, OR (95% CI)^a^
Action plan engagement^b^		
None	51 (29.5)^c^	1 [Reference]
Limited	29 (16.8)	0.18 (0.06-0.52)
Full	93 (53.8)	0.36 (0.15-0.88)
Action plan completion^b^		
None or partly	85 (49.1)^c^	1 [Reference]
Mostly	46 (26.6)	0.52 (0.24-1.12)
All	42 (24.3)	0.55 (0.25-1.20)

Abbreviation: OR, odds ratio.

^a^ Adjusted by sex, current age, years after cancer diagnosis, health insurance status, history of survivorship clinic attendance, body mass index, and undertreated condition.

^b^ Based on study clinician rating after 4-month study check-in.

^c^ Participants who did not schedule a 4-month check-in (n = 45) were classified as not engaged and without action plan completion; these 45 include 25 participants who never scheduled a baseline session; 22 participants who did not complete the 4-month check-in completed the 1-year follow-up assessment.

When PCC records were reviewed, records belonging to intervention participants had modestly improved documentation of key elements relating to CVD risk and survivorship care compared with controls (eTable 5 in Supplement 2). The intervention was associated with increased documentation of participants with cardiotoxic chemotherapy (12.3% improvement vs 1.9%; *P* = .03), being at increased CVD risk (14.8% improvement vs 0.9%; *P* = .002), and presence of an SCP within the medical records (20.2% improvement vs −1.4%; *P* < .001).

## Discussion

Although the CHIIP intervention was not associated with reduced CVD risk factor undertreatment compared with an enhanced care control condition that received results alone, 26.0% of intervention participants and 30.2% of control participants had a reduction in undertreated CVD conditions after 1 year. This improvement was seen for hypertension, dyslipidemia, and glucose intolerance. Because of participants’ prior anthracycline chemotherapy and/or chest radiotherapy exposures, all are considered as having stage A heart failure (ie, at risk for heart failure but without symptoms or structural and/or functional disease), for whom controlling hypertension, dyslipidemia, and glucose intolerance are long-standing recommendations.^28^ In addition, based on survivor-specific prediction models, many participants also have an increased risk of ischemic heart disease, with a 10% or greater predicted risk of serious disease by 50 years of age, with risk further exacerbated by the presence of undertreated CVD risk factors.^4^

In designing CHIIP, we wanted to approximate a standard clinical encounter where survivorship care is often delivered by specialized APPs, who typically see patients annually, at most. The intervention built on the experiences of 2 other randomized trials conducted within CCSS.^37,38^ Evaluation of Cardiovascular Health Outcomes Among Survivors (ECHOS)^37^ featured an advanced practice nurse–led counseling intervention (2 telephone calls) that addressed barriers to cardiomyopathy screening, supplemented by provision of an SCP and tailored screening recommendations to both intervention and control groups. After 1 year, 52% of intervention participants had received screening echocardiograms vs 22% of controls. EMPOWER^38^ tested a 1-time motivational interview with supplemental educational materials to address barriers to breast cancer screening and found that after 1 year, the intervention was associated with a 33% rate of screening mammograms vs 18% in attention controls. Both studies provided materials for participants to give their PCCs but did not engage directly with PCCs.

Although CHIIP’s intervention was of similar intensity to these 2 studies,^37,38^ some distinctions may explain the different results. Activating PCCs to order recommended screening studies may differ from achieving differential outcomes in physiologic end points, as CHIIP attempted to do. More frequent and/or dynamic interactions with both survivors and PCCs may be required to achieve greater rates of CVD risk factor control. In qualitative interviews with participants, some expressed a desire for more support from the study clinician beyond a single booster session.^39^ Adding other strategies such as motivational interviewing may enhance the intervention, as participants who were more engaged with their self-management plan had greater improvement. Another distinction to ECHOS and EMPOWER was that CHIIP sent all study materials directly to participants’ PCCs. For control participants, this included copies of their home assessment, including clear notation of abnormalities like a standard clinical report. Simply providing CVD screening for controls and requesting that their PCCs follow-up may explain the substantial reduction in undertreatment seen in that group. A true usual care control group without access to these screening results would have clarified this potential benefit but was deemed unethical.

The US Preventive Services Taskforce (USPSTF) recommends annual hypertension screening in adults 40 years or older.^40,41^ Less frequent screening every 3 to 5 years is appropriate for younger adults unless they are thought to be at increased hypertension risk (eg, overweight, history of borderline blood pressures). Prior USPSTF recommendations also included consideration of routine lipid screening in men 35 years or older and women 45 years or older.^42^ Younger adults were recommended for screening if they were thought to be at increased risk for coronary heart disease. For diabetes, the USPSTF recommends screening in adults aged 35 to 70 years who are overweight, having recently lowered the age limit of 40 years.^43^ Given our findings that most adult survivors of childhood cancer who are at increased CVD risk due to their cancer history lack such documentation in their medical records, it was not surprising that many study participants did not have specific CVD risk factor screening, given their younger age and lack of perceived risk factors. Although the CHIIP intervention was not more effective compared with the control condition in reducing undertreatment, it is possible the improved CVD risk documentation seen in the intervention group may contribute to better outcomes longer-term. We previously found that documentation of CVD risk was associated with a greater likelihood of having recommended cardiac screening.^14^

### Limitations

The trial recruited participants from a long-standing cohort study. Although CCSS is reflective of the demographic characteristics of children treated for cancer from the 1970s to 1990s,^44^ contemporary patients with pediatric cancer are more racially and ethnically diverse. In particular, trial eligibility was limited to English speakers, which excludes more vulnerable populations from minority racial and ethnic groups. It is also possible that results could differ among survivors with less access to primary care, including those living in more rural areas. Due to logistical considerations, we only approached participants living in closer proximity to selected metropolitan areas. Last, although the trial did not meet its prespecified accrual goal, given the degree of improvement in undertreatment seen in the control group, even a fully accrued trial would not have had sufficient power to detect a significant difference. As such, we can only say that the nonsignificant primary result is inconclusive rather than evidence of null effect or noninferiority.

## Conclusions

In this randomized clinical trial of adult survivors of childhood cancer, we found that provision of recommended CVD risk factor screening for hypertension, dyslipidemia, and glucose tolerance was associated with a substantial reduction in undertreatment of these risk factors after 1 year. While the addition of an SCP with personalized health information and 2 counseling sessions did not further reduce undertreatment, it was associated with increased documentation of CVD risk in medical records. Our results also suggest that additional strategies to mitigate CVD risk in high-risk survivors (eg, motivational interviewing, additional sessions) should be examined in the future.

## Notes

Supplement 1.
Trial Protocol

Supplement 2. **eTable 1.** US Metropolitan Areas Used for Study Recruitment **eTable 2.** Distribution of Undertreated Conditions Based on Home Visit Measurements by Randomization Status, Corresponding to Estimates Shown in Main Figure 1 **eTable 3.** Differences in Outcomes by Randomized Group, and Within Each Group at 1-Year Follow-Up vs Baseline **eTable 4.** Odds of Undertreatment Among Trial Participants After 1 Year in Relation to Change in Self-Reported Self-Efficacy and Baseline Multidimensional Health Locus of Control (MHLC) Domains **eTable 5.** Changes in Primary Health Care Provider Documentation After 1-Year Follow-Up by Randomization Status (293 of 347) **eAppendix.** Cancer Treatment Summary and Survivorship Care Plan

Supplement 3.
Data Sharing Statement
