# Supplementary material for: Counseling and Cardiovascular Disease Risk Factor Control in Long-Term Cancer Survivors: A Randomized Clinical Trial
Source: JAMA Netw Open. 2026 Feb 5;9(2):e2555863. doi: 10.1001/jamanetworkopen.2025.55863 (PMC12878413; doi:10.1001/jamanetworkopen.2025.55863)
Supplement: Supplement 1. — Trial Protocol [file jamanetwopen-e2555863-s001.pdf]

FRED HUTCHINSON CANCER RESEARCH CENTER
UNIVERSITY OF WASHINGTON SCHOOL OF MEDICINE
SEATTLE CHILDREN'S

7/10/20

Title of Protocol:
Communicating Health Information and Improving Coordination with Primary Care (CHIIP) Study

Investigators List:		
Eric J. Chow, MD, MPH	Associate Professor, FHCRC, Associate Professor of Pediatrics, UW	(206) 667-4630
Gregory T. Armstrong, MD, MSCE	Member, St. Jude Children's Research Hospital	
Laura-Mae Baldwin, MD, MPH	Professor of Family Medicine, UW	
Melissa Hudson, MD	Member, St. Jude Children's Research Hospital	
Lisa Johnson, PhD	Senior Staff Scientist, FHCRC	
Paul Nathan, MD, MSc	Professor of Pediatrics, University of Toronto	
Kevin Oeffinger, MD	Professor of Medicine, Duke University	
Charles Sklar, MD	Professor of Pediatrics, Memorial Sloan-Kettering Cancer Center	
Karen Syrjala, PhD	Professor, FHCRC	
Yutaka Yasui, PhD	Member, St. Jude Children's Research Hospital	

SPONSOR: National Cancer Institute

Coordinating Center:		
Fred Hutchinson Cancer Research Center		
Additional Performance Sites:		
St. Jude Children's Research Hospital	Gregory T. Armstrong, MD, MSCE	(901) 595-6078

PROTOCOL SYNOPSIS

Protocol Title	CHIIP Study (Communicating Health Information & Improving coordination with Primary care)
Protocol No.	FH IRB Files 8543 and 8543-A and Clinical Trial NCT03104543
Sponsor	National Cancer Institute
Trial Type	Randomized clinical trial of a counseling/educational intervention
Clinical Indication	Survivors of childhood cancer are known to be at higher risk of developing premature, serious cardiovascular disease compared with the general population. Hypertension, dyslipidemia, and diabetes increase this risk beyond that attributable to one's original cancer therapy exposures. Research has shown that childhood cancer survivors also have a high burden of underdiagnosis and undertreatment of these potentially modifiable conditions.
Study Objectives	1. To determine the prevalence of underdiagnosis and undertreatment of common cardiometabolic conditions (i.e., hypertension, dyslipidemia, diabetes) in survivors of childhood cancer at high-risk of future serious cardiovascular disease.<br>2. Among survivors who are found to be underdiagnosed or undertreated, to determine (via randomized clinical trial) the efficacy of a counseling/educational intervention to improve control of these cardiometabolic conditions.<br>3. Determine barriers on among survivors enrolled on the randomized trial and their primary healthcare providers that contribute to undertreatment of the study's targeted cardiometabolic conditions.
Study Design	Aim 1 is an observational study. Aims 2 and 3 are based on a randomized clinical trial design.
Population	Participants in the existing Childhood Cancer Survivor Study cohort who are classified as high-risk for future cardiovascular disease based on their demographic and cancer therapy exposures.
Primary Endpoints	Aim 1: Prevalence of underdiagnosis and undertreatment of hypertension, dyslipidemia, and diabetes.<br>Aim 2: Probability of having an undertreated condition
Secondary Endpoints	Aim 3: Determine whether intervention and control arms differ in terms of survivor knowledge, self-efficacy, and medication adherence, as well as differences in their providers' knowledge and self-efficacy related to the care of childhood cancer survivors.
Type of control	Delayed intervention control (i.e., can choose to receive the intervention at the end of the study period).
Trial Blinding	Study staff performing home visits and study statisticians will be blinded. Participants and study staff delivering the counseling intervention cannot be blinded given the nature of the study.
Treatment Groups	Group A: upfront intervention group (30 minute counseling session focused on survivorship care plan, personalized action plan; 15 minute booster session at 4 months)<br>Group B: delayed intervention control group (30 minute counseling session focused on survivorship care plan, personalized action plan)
Treatment Schedule	Baseline: educational materials (survivorship care plan, personalized action plan, and 30 minute counseling intervention<br>4 months: 15 minute booster session
Efficacy Assessments	Baseline: patient questionnaire and 1st home visit<br>4 months: action plan scoring by study staff<br>12 months: patient questionnaire and 2nd home visit
No. subjects	Anticipate 800 for Aim 1, and project that ~480 maybe eligible to participate on Aims 2 and 3.
Estimated duration	5-years
Duration of Participation	1-year interval between initial survey and home visit and follow-up survey and home visit.

Table of Contents

1.0 GENERAL INFORMATION 5
2.0 INTRODUCTION 5
2.1 Introduction 5
2.2 Clinical Data to Date 8
2.3 Risks/Benefits 10
3.0 OVERVIEW OF CLINICAL TRIAL 11
3.1 Study Objectives 11
3.2 Study Population 12
3.3 Study Design 12
3.4 Estimated Accrual 13
3.5 Sponsor/Funding Source 13
4.0 SAFETY CONSIDERATIONS 13
5.0 SUBJECT ELIGIBLITY 14
5.1 Inclusion Criteria 14
5.2 Exclusion Criteria, applicable to all participants 15
6.0 SUBJECT RECRUITMENT & REGISTRATION 15
6.1 Recruitment 15
6.2 Registration 16
7.0 TREATMENT PLAN 17
7.1 Treatment Plan Overview 17
7.2 Assignment of Study Group 17
7.3 Intervention Group 18
7.4 Delayed Intervention Control 18
7.5 Interaction with Primary Healthcare Providers 18
7.6 Duration of Therapy and Follow-Up 19
8.0 STUDY PROCEDURES 20
8.1 Baseline Questionnaire 20
8.2 First Home Visit 21
8.3 Baseline Counseling Session (Intervention Group) 21
8.4 Baseline Return of Results (Control Group) 22
8.5 Handling of Critical Results 22
8.6 Four-month Booster Session 23
8.7 Second Questionnaire and Home Visit 23
8.8 Primary healthcare provider measurements 24
8.9 Materials returned to participants and primary healthcare providers 24
8.10 Remote blood pressure monitoring pilot 25

9.0 OFF STUDY CRITERIA 26
10.0 ADVERSE EVENTS 26
10.1 Expected Adverse Events 26
10.2 Monitoring and Recordings Adverse Events 27
11.0 DATA AND SAFETY MONITORING PLAN 27
12.0 DATA MANAGEMENT/CONFIDENTIALITY 27
13.0 STATISTICAL CONSIDERATIONS 27
13.1 Aim 1 Analytic Plan 28
13.2 Aim 2 Analytic Plan 28
13.3 Aim 3 Analytic Plan 29
13.4 Ethnic and Gender Distribution Chart 32
14.0 REFERENCES 33
15.0 APPENDICES 37

1.0 GENERAL INFORMATION

Children and adolescents diagnosed with cancer now have on average >80% 5-year survival. However, premature cardiovascular (CV) disease has become the leading non-cancer cause of late mortality among childhood cancer survivors. Our existing work has shown that traditional CV risk factors such as hypertension, dyslipidemia, insulin resistance/diabetes remain very important, by increasing (in synergistic fashion) the risk of major CV events such as ischemic heart disease and heart failure. However, the existing research has been limited by misclassification of CV risk factor status (i.e., when defined by self-report or medication usage alone), including issues with both underdiagnosis (i.e., people with these risk factors present but who are unaware) and uncertainty of disease control (i.e., potential undertreatment).

This NCI-funded proposal will utilize the largest, best characterized childhood cancer survivor cohort in the world, the Childhood Cancer Survivor Study (CCSS; n=24,466). The CCSS, known to study participants as the "Long Term Follow-Up [LTFU] Study", is a National Cancer Institute (NCI) funded resource with its data coordination center at St. Jude Children's Research Hospital and its statistical center at FHCRC.[1] CCSS promotes the development of "ancillary" studies that obtain supplemental funding to extend cohort research beyond what the parent grant can accomplish. Thus, for the proposed CHIIP Study, we will use CCSS-derived validated risk prediction algorithms to select CCSS survivors at high risk of serious heart disease (n~800) based on past cancer treatment exposures. Among these 800 survivors, we propose to determine the magnitude of <u>underdiagnosis</u> and <u>undertreatment</u> of hypertension, dyslipidemia, and diabetes via in-person (home-based) measurements supplemented by medical record review. We predict that around 60% of survivors (n~480) will be underdiagnosed or undertreated with respect to one of these three CV risk factors. *Survivors who are underdiagnosed or undertreated will then be eligible to participate in a <u>1-year long randomized controlled trial</u>, where we will measure the efficacy of an Institute of Medicine (IOM) recommended personalized survivorship care plan (SCP) emphasizing CV risk, supplemented by a remotely delivered clinician-led self-management counseling intervention, to improve control of these three CV risk factors (i.e., reduce rates of undertreated hypertension, dyslipidemia, and diabetes).* Survivors randomized to the control arm can receive the intervention on a delayed basis. Finally, our proposal seeks to better understand barriers among survivors and their primary healthcare providers that contribute to CV risk factor undertreatment.

Knowledge derived from this study will improve the assessment and treatment of important CV risk factors in this high risk population. The proposed intervention, if successful, will be disseminable and low cost, and will have the potential to improve health and reduce mortality in these younger adults who live the majority of their lives as cancer survivors at increased risk of serious CV disease. The protocol described below is classified as clinical research and will be conducted in compliance with the Fred Hutchinson Cancer Research Center / Cancer Consortium's approved policies/procedures, and in compliance with all associated Federal regulations.

2.0 INTRODUCTION

2.1 Introduction

There are nearly half a million survivors of childhood cancer estimated to be living in the United States.[2] Premature CV disease is a leading contributor to late morbidity and mortality in this population.[3-7] Cohort studies from North America and Europe, including the CCSS, have consistently shown that

survivors have a 5+ fold increased risk of serious CV morbidity or mortality vs. the general population, corresponding to ~5% cumulative incidence by age 45 years.[3-11] Among survivors that have been exposed to cardiotoxic cancer treatments (i.e., anthracyclines and chest radiotherapy), this risk can be markedly greater (**Table 1**).[6,7] Demographic characteristics such as age at treatment and gender, and off-target and indirect effects of both radiotherapy and chemotherapy may also affect CV health.[6,7,12-14]

TABLE 1. *Cardiovascular (CV) risk group outcomes among the original CCSS cohort through age 50.*

Serious CV event	Predicted low risk*		Predicted high risk*	
	No. events / no. at risk	Cumulative incidence / relative risk (RR) vs. siblings	No. events / no. at risk	Cumulative incidence / relative risk (RR) vs. siblings
Ischemic heart disease	73 / 8801	2.3% / RR=2.3, p<0.001	89 / 764	19.9% / RR=17.8, p<0.001
Heart failure	18 / 5197	1.0% / RR=1.8, p=0.11	108 / 2059	12.4% / RR=41.5, p<0.001

*Risk prediction for 5-yr survivors (n=13,060) through age 50, based on sex, diagnosis age, anthracycline and chest radiotherapy doses.[12,15] Area under the curve [AUC]/C-indices for these models ranged 0.70-0.76 (CCSS), and 0.66-0.82 (external validation cohorts).

Multiple studies in childhood and adult cancer survivors also have shown that even after considering treatment exposures, the presence of conventional CV risk factors such as hypertension, diabetes, and dyslipidemia are important, and may further increase the risk of serious CV disease *in more than additive fashion.*[7,16,17] Among CCSS participants, hypertension was associated with significantly increased relative excess risks due to interaction [RERI] for ischemic heart disease (after chest radiotherapy) and heart failure (after anthracyclines; **Figure 1**); RERI was also significantly increased for dyslipidemia and diabetes.[7] Furthermore, survivors predicted to be at high risk for ischemic heart disease and heart failure, on the basis of their cancer treatment exposures alone, also had higher self-reported rates of hypertension, dyslipidemia, and diabetes vs. survivors predicted to be at low risk (**Table 2**). Among childhood cancer survivors, the development of these conditions also tends to occur at younger ages compared with siblings or the general population.[18-21] Given the relatively young age of onset of these conditions that occur more typically in older adults, and the limited knowledge of cancer survivor-specific screening guidelines among general practitioners,[22,23] most high risk survivors likely do not receive recommended CV screening studies.[24-26] *Thus, there is a compelling rationale to develop interventions for this high risk population designed to target these modifiable CV risk factors.*

FIGURE 1. *Relative risk of serious CV outcomes in CCSS per treatment exposures & hypertension status.*[9]

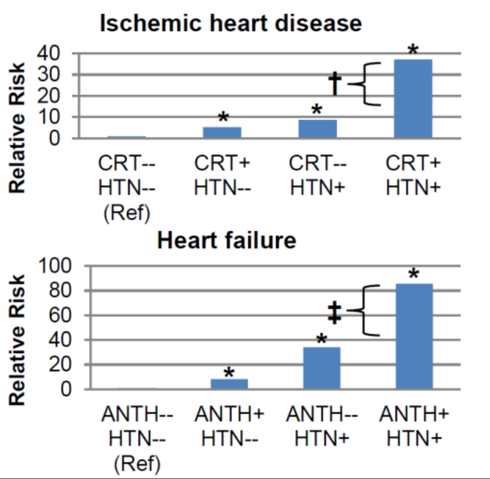

ANTH[racycline]; CRT, chest radiotherapy; HTN, hypertension. Relative excess risk due to interaction (RERI) between treatment exposure & hypertension for these 2 outcomes: †RERI=24 (95% CI 12-40), ‡RERI=45 (95% CI 17-106). *RERI>0 indicates that interaction was more than additive.* *P<0.01 vs. referent.

A frequent <u>limitation</u> of many studies that have examined the relationship between hypertension, dyslipidemia, diabetes, and subsequent more serious outcomes in survivors of childhood cancer, including some of our own work, is reliance on self-report and/or the use of medications as surrogates for these risk factors, in lieu of physiologic or more objective clinical data. Where such data have been available, they often have been collected retrospectively, in a non-standardized fashion, or are only available in cross-sectional analyses with either relatively limited sample sizes, recruited from a single

center, and/or a focus on a single cancer type, all of which limit generalizability.[5,19,20] However, these studies support the hypothesis that <u>underdiagnosis</u> of these CV risk factors is common among survivors.

Also unanswered is the degree to which survivors known to have hypertension, dyslipidemia, or diabetes are adequately treated (<u>undertreatment</u>). We are unaware of studies that have attempted to examine CV risk factor undertreatment among childhood cancer survivors, and such studies are rare among survivors of adult cancers.[27-31] Our prior research showed that survivors with these CV risk factors, defined on the basis of medication use, were at significantly greater risk of more serious CV events (e.g., ischemic heart disease, heart failure) vs. other survivors.[7,17] Thus, we hypothesize that survivors may be significantly undertreated even when diagnosed. The drivers of undertreatment may be related to healthcare providers not intervening sufficiently and/or non-adherence to appropriate interventions among survivors themselves.[27,32,33]

TABLE 2. *Prevalence of selected CV risk factors by 10-year age groups, stratified by CV-risk status* among the original CCSS cohort (n=13,060) and a sibling comparison group (n=4,023).*

	Ischemic heart disease risk model	Heart failure risk model
Hypertension[†]		
Dyslipidemia[†]		
Diabetes[†]		

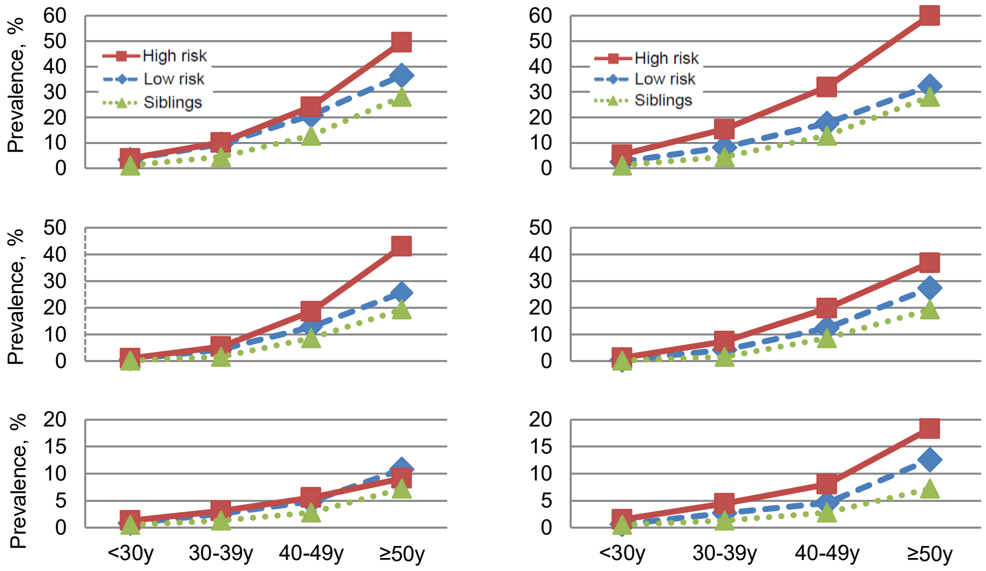

Prediction AUC (C-index)[‡]	Ischemia	Heart failure
with the above risk factors	0.76 (0.74)	0.76 (0.78)
without the 3 risk factors	0.74 (0.73)	0.75 (0.77)
Difference in model fit	p=0.01 (p=0.07)	p=0.04 (p=0.03)

*Low and high risk groups for ischemic heart disease and heart failure based on prediction models developed for 5-yr cancer survivors (see Table 1). [†]Defined as those who reported being diagnosed by a physician for the condition(s) and who took specific medications to treat that condition(s) for >1 month or for ≥30 days in a 1 year period during the previous 2 years. [‡]Prediction AUC (C-indices) based on 10,521 survivors with CCSS questionnaire data, and incorporates presence of CV risk factor information ([†]) present at baseline risk assessment (age 26).

The study's intervention will feature an IOM-recommended survivorship care plan [SCP], which is designed to promote knowledge/awareness of personal health risks among survivors and to help disseminate that information to primary healthcare providers.[34] Prior research has shown that >80% of adult survivors of childhood cancer are followed by primary care providers,[25,26,35] and that while the majority of internists and family practitioners report caring for childhood cancer survivors, receipt of a SCP remains uncommon and the vast majority of surveyed providers do not report familiarity with long-

term follow-up guidelines for childhood cancer survivors.[22,23] Our SCP intervention also will be enhanced by the use of recently developed and validated individual CV risk predictors our group has created specifically for childhood cancer survivors (**Tables 1, 2**). Although the SCP is by itself a tool to foster self-management, we will further supplement it with well-established, focused chronic disease self-management strategies now being applied to improve coordination of cancer survivorship care (**Figure 2**).[36-39] Collection of directly measured data also offers an opportunity to further refine risk prediction in the future, beyond using self-report alone (**Table 2**).

In summary, our proposal will systematically assess the magnitude of underdiagnosis and undertreatment, along with contributing survivor- and provider-specific barriers, among adult-aged CCSS participants predicted to be at high risk of future serious CV disease. Results from this proposal may significantly advance our understanding of CV disease risk among childhood cancer survivors of all ages, *given that many of the cancer treatments CCSS participants received remain in common use today.*[40,41] If successful, our IOM-based personalized intervention will increase the proportion of survivors and healthcare providers who are aware of current screening guidelines, who receive/deliver more appropriate CV treatment, and who adhere to these guidelines and treatments. The cumulative effect will be a mitigation of survivors' long-term CV risks.

2.2 Clinical Data to Date

FIGURE 2. *Survivorship self-management model (adapted from Schulman-Green, McCorkle, and colleagues.[46]*

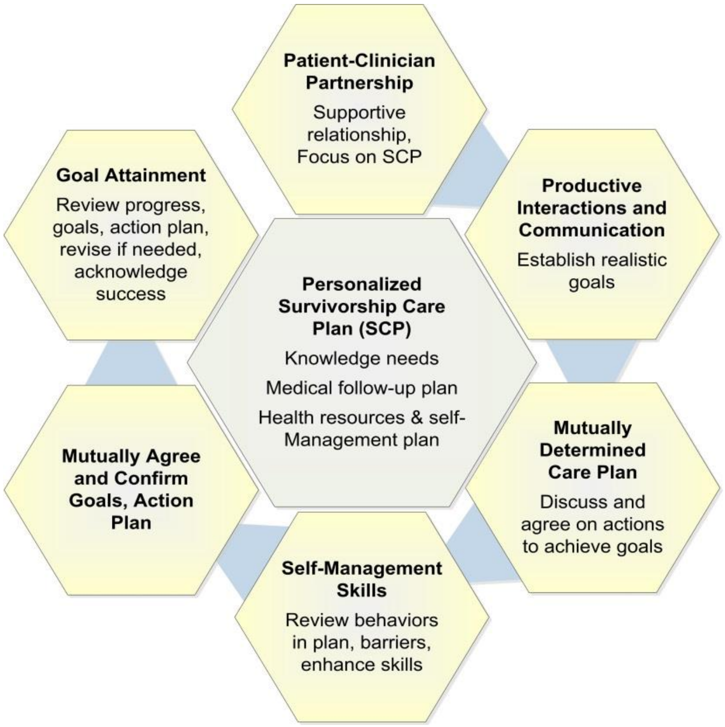

The home sampling methodology proposed is based on the NHLBI-supported Women's Health Initiative's (WHI) recently completed Long Life Study, where women (age ≥63 years) underwent home-based sample collection using Examination Management Services, Inc. (EMSI). Among 14,000 eligible women, 66% consented, and 85% of those who consented had a biospecimen collected with excellent results (≥95% successful collection). With WHI investigator input, CCSS conducted an EMSI pilot study from Fall 2014 to Spring 2015, approaching 200 randomly selected CCSS participants living within a 50-mile radius of 2 metropolitan areas (Minneapolis & Philadelphia) to complete a home visit featuring a 12-page questionnaire, followed by physical measurements and a fasting blood draw. Participants received a $50 gift card <u>after</u> study completion and prompt notification (<72 hours) of any clinically meaningful lab results (standard metabolic panel, lipid profile, hemoglobin [Hb] A1c). After 6 months, 60% of participants had consented (11% active refusals). The median age of participants was 43 years (range 30-57). Among those who consented, 90% completed home-based data collection (all with viable samples, n=107) as of April 2015. Although women were more likely to participate than men (58% vs. 42%), participation did not differ significantly by race/ethnicity, age, or treatment era. *Overall,*

the CCSS results approximate those from WHI, despite having a population that is half men, who generally have lower participation rates than women.[42] Given concerns that more urban CCSS participants may differ from others, we found that those who lived <50 vs. >50 miles from an existing CCSS center (all major metropolitan areas) were similar with respect to demographic and treatment characteristics, except that racial/ethnic minority status was more common among participants residing <50 miles (18% vs. 11%).

FIGURE 3. *Yield of directly measured data in relation to self-report among randomly selected CCSS participants (n=175).*

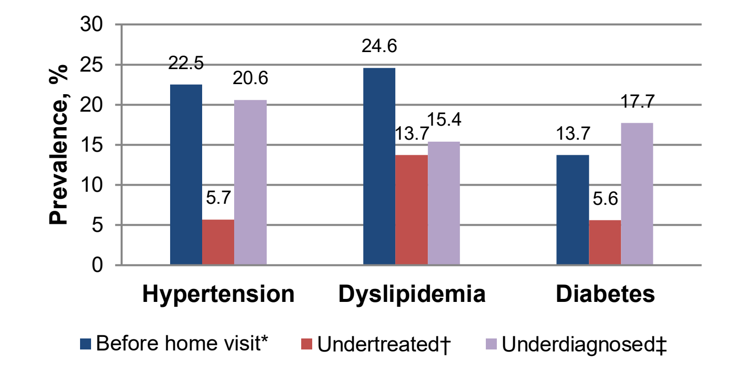

*Per self-report. †Among those with self-reported condition if home sampled value was beyond standard therapeutic range. ‡No prior self-reported history of respective condition with abnormal value.

Direct measurement of participants from the EMSI pilot study plus data from randomly selected CCSS participants directly measured in 2013 found that abnormal blood pressure, fasting lipid, or blood glucose values were common (median age 42; 45% male). 37% of participants had ≥1 value (blood pressures ≥140/90 mmHg, LDL cholesterol ≥160 mg/dL or triglyceride ≥150 mg/dL, blood glucose ≥126 mg/dL or HbA1c ≥6.5%) meeting commonly accepted clinical standards for therapeutic intervention *(i.e., lifestyle prescription and/or medication management).* This included 23% of survivors who did not have a self-reported history of any of these 3 conditions (i.e., potential underdiagnosis). If thresholds included higher pre-hypertension values (≥130/85 mmHg) and pre-diabetes (glucose ≥100 mg/dL or HbA1c ≥5.7%), the prevalence of those with an abnormal value increased to 50% (**Figure 3**). Specifically, among 52 high CV risk survivors, ~60% had an abnormal CV measurement, with the prevalence of individual risk factors ranging 20-40%. Among those with abnormalities, ~40% were undertreated (prevalence of individual undertreated conditions 5-20%) and ~80% had a potentially underdiagnosed condition (prevalence of individual underdiagnosed conditions 15-30%). 20% of survivors also had underdiagnosis of 1 condition and undertreatment of another condition. *These data support our hypotheses that both underdiagnosis and undertreatment of CV risk factors are common among adult-aged survivors of childhood cancer, and that previously published rates based on self-report, particularly if restricted to medication use, underestimate and misclassify the true burden of these risk factors.*

The randomized intervention component of the CHIIP study will be based upon past CCSS intervention studies that incorporated distance-based delivery of SCPs (led by Drs. Oeffinger & Hudson, both co-investigators).

PROJECT VISION (PI: Oeffinger; Livestrong; 2003-05) enrolled 62 (86% participation rate) adult-aged Hodgkin lymphoma survivors at increased risk for either breast cancer or cardiomyopathy to test the acceptability of an IOM-based SCP supplemented by a study website and an effort to engage participants' primary care providers.[43] 92% of participants completed a *6-month* post-intervention survey; 75% remembered receiving, reading, and understanding the SCP. Among participants, prior knowledge of mammogram and echocardiogram screening recommendations was low (32% and 12% respectively). During the 6-month study period, 41% and 20% of eligible respondents obtained a mammogram and echocardiogram, respectively, with another 35% (for both tests) planning to do so

within the next 6 months. *As such, these data guide our planned intervention follow-up period of 12 months between assessments.* Similar to other studies,[44] website usage was limited among these young adult survivors (29% visit rate). Primary care providers demonstrated limited engagement: only 19% of providers volunteered to participate; none visited the study website or contacted the study team.[43]

The ECHOS trial (co-PI: Hudson; R01 CA136912; 2009-13) extended the methods in VISION to directly test the efficacy of a SCP supplemented by 2 nurse practitioner-led phone self-management counseling sessions on cardiomyopathy screening (vs. mailed SCP alone).[45] *After 1-year,* the randomized intervention group had higher rates of obtaining recommended echocardiograms per self-report validated by medical records (52% vs. 22% SCP alone [p<0.001]; RR 2.3, 95% CI 1.7-3.1). The intervention group also was less likely to report lack of physician recommendation as a reason for not obtaining an echocardiogram, suggesting that the counseling further enhanced communication between recipients and their primary care provider beyond what a SCP alone would have accomplished.[46] The study had an 87% retention rate at 1-year follow-up (n=411 of 472).[45]

2.3 Risks/Benefits

The proposed home visits and biosample and data collection itself pose minimal risk to participants. Anthropometric measurements are all non-invasive. There would be a blood draw (thus potential for brief mild pain) associated with any home visit (1 to 2 over the course of the study for any given participant). The study team will ensure that the amount drawn at any visit is safe from a blood volume standpoint. It is possible, but unlikely, that some survey questions may cause participants to feel uncomfortable. All instruments proposed have been previously used in other research.

For participants selected to participate in the randomized intervention trial, it is possible that receipt of clinical lab results may cause some participants to feel uncomfortable or anxious. Similarly, receipt of a SCP with an individual’s predicted cardiovascular risk may cause anxiety or discomfort. We will be monitoring self-reported anxiety and depression as part of our study. Nevertheless, we feel that providing such information, even when unexpected, may be important and have important future health consequences, as the research data to be provided to participants are all results with clinical interpretation and significance. For participants on the intervention arm, they will get a telephone or web video session by which any results are given and explained to them by a survivorship trained clinician, approximating a normal clinical exchange. Participants on the control arm will only get a letter summarizing clinically relevant results, and will be encouraged to discuss any abnormal results with their primary healthcare provider. The study has in place, a process by which critical test results will be reported to the study team in real time, in which case, the PI or his designee, can notify the participant more quickly to seek medical follow-up. This process is similar to and adapted from that used by the CDC-sponsored NHANES for their in-person mobile assessments. For participants (either intervention or delayed control) with clinically actionable results but who have no current healthcare provider, the study team has access to resources to aid participants in finding providers in their area who have expertise in treating survivors of childhood cancer. This will be clearly explained on their results letter.

Control participants will get access to the SCP with personalized recommendations and a self-management counseling session with formal written summary “action plan” following the 2nd home visit if they chose to. Finally, all healthcare providers designated by the study participant will also receive a copy of the same clinically relevant study test results. Providers of intervention study participants also will receive a copy of the SCP with personalized recommendations following both home visits. SCPs are IOM-recommended documents now felt to be integral to cancer care, but whose efficacy in modifying

clinical endpoints has not been rigorously studied. Prior CCSS and other research also have shown that most cancer survivors have not previously received a SCP.

3.0 OVERVIEW OF CLINICAL TRIAL

3.1 Study Objectives

We propose to determine the magnitude of hypertension, dyslipidemia, and diabetes underdiagnosis (i.e., people with these risk factors but unaware) and undertreatment (i.e., previously diagnosed but not achieving standard treatment goals) among a national sample of childhood cancer survivors. We will then test, in a randomized controlled design, the efficacy of an Institute of Medicine (IOM) recommended personalized survivorship care plan (SCP) intervention designed to improve control (i.e., reduce undertreatment) of these three CV risk factors. SCPs contain a summary of one's past cancer treatment and an evidence-based follow-up plan. They are devised to bridge the knowledge gap regarding cancer treatment late effects among both survivors and primary healthcare providers. However, despite endorsement of SCPs by the IOM and other groups, few studies have examined the efficacy of SCPs in modifying clinical endpoints. Finally, we will attempt to understand barriers to CV risk factor undertreatment among both survivors and healthcare providers – an understudied area among cancer survivors. home) assessments.

3.1.1 Primary Aim 1.

Determine the prevalence of underdiagnosis and undertreatment of conventional CV risk factors (i.e., hypertension, dyslipidemia, and diabetes) among CCSS participants predicted to be at high risk (n~800) for future serious CV disease (i.e., ischemic heart disease, cardiomyopathy/heart failure) on the basis of their original cancer treatment exposures (e.g., chest radiotherapy, anthracycline doses).

- *Hypothesis: At the initial home-visit, ~60% (n~480) will have a blood pressure, lipid, and/or glucose value that meets clinical thresholds for intervention. Among those with abnormalities, 40% will have a known pre-existing CV risk factor diagnosis but are undertreated, and 80% will be newly diagnosed.*

3.1.2 Primary Aim 2

Among survivors found to be underdiagnosed or undertreated (Aim 1), in a randomized controlled design, compare changes in blood pressure, lipid, and blood glucose values from baseline to 1-year between those receiving the intervention (providing clinical results and survivorship care plans [SCPs] to participants and their healthcare providers, supplemented by clinician-led remote counseling sessions with participants to review SCP contents and teach CV risk factor self-management strategies) vs. control (providing clinical results without SCP to participants and their healthcare providers; with delayed access to the intervention).

- *Hypothesis: At the 1-year follow-up home visit, survivors randomized to the intervention arm will have a lower probability of having an undertreated CV risk factor compared with survivors in the control arm.*

3.1.3 Primary Aim 3

Determine barriers among (Aim 2) survivors (at baseline and 1-year: knowledge of

past cancer treatment, self-efficacy, health-related attitudes, medication adherence) and their primary healthcare providers (at 1-year only: knowledge and self-efficacy towards childhood cancer survivorship care) that contribute to undertreatment of hypertension, dyslipidemia, and diabetes.

- Hypothesis 1*: At baseline, underdiagnosis and undertreatment will be associated with lower knowledge, self-efficacy, and medication adherence, as well as a "self-controlling" health attitude among survivors.*
- Hypothesis 2*: At 1-year follow-up, intervention-arm participants will report improved knowledge, self-efficacy, and medication adherence vs. controls; healthcare providers who received the SCP will report improved knowledge and self-efficacy towards CV risk and survivorship care vs. providers of controls.*

3.2 Study Population

The study would seek to enroll ~800 current participants of the Childhood Cancer Survivor Study (CCSS) cohort, an existing NIH-funded resource study with >24,000 cohort members that has been actively funded for >20 years. The study would secondarily also survey participants' designated primary healthcare provider regarding knowledge and self-efficacy related to care of childhood cancer survivors.

3.3 Study Design

The study's first primary aim will be answered by recruiting a cross-sectional sample of predicted high CV risk patients. Those who meet eligibility criteria will then be randomized to a controlled intervention with delayed intervention controls to answer the study's second and third primary aims.

3.3.1 **Primary Aim 1 Endpoint:** Prevalence of underdiagnosis and undertreatment of the following target CV conditions. *Underdiagnosis* defined for each as:

- Hypertension: Joint National Committee (JNC7/8)[47,48] considers systolic 120-139 and diastolic pressures 80-89 mmHg to be suggestive of pre-hypertension; *lifestyle intervention* is recommended.[47,49] Hypertension is suspected if systolic ≥140 or diastolic ≥90 mmHg, and in addition to lifestyle modification, *pharmacologic treatment* is recommended for adults <60 years (treatment threshold ≥150 systolic if ≥60 years).[48] We will ascertain the prevalence of pre-hypertension and hypertension; both may be eligible for Aim 2. *However, with the introduction of the 2017 ACC/AHA hypertension guidelines, the classification system has shifted such that systolic ≥130 or diastolic ≥80 mmHg are now considered Stage 1+ hypertension (with treatment threshold reduced accordingly).[50] As such, with our 12/2017 protocol modification, we will adopt these slightly more stringent thresholds for eligibility and drop the prior "pre-hypertension" category.*
- Dyslipidemia: American Heart Association and American Academy of Pediatrics recommend intervening *(lifestyle modification first; if unsuccessful, consider pharmacologic therapy)* among childhood cancer survivors for LDL ≥160 mg/dL or fasting triglyceride ≥150 mg/dL.[51] As fasting blood draws are not as commonly done in most primary care settings now for initial screening, for non-fasting (<10 hours) samples, a triglyceride ≥200 mg/dL is considered high. This study will use these cut-points to define dyslipidemia. These thresholds were largely in-line with

those defined by a recent NIH-sponsored expert panel,[52] and largely similar to prior NCEP Adult Treatment Panel recommendations.[53]

- Diabetes: American Diabetes Association classifies fasting blood glucose 100-125 mg/dL or HbA1c 5.7-6.4% as suggestive of impaired glucose tolerance (pre-diabetes). Similar to dyslipidemia, as fasting blood draws are not as commonly done in most primary care settings now for initial screening, for non-fasting (<8 hours) samples, blood glucose 140-199 mg/dL will be classified as suggestive of pre-diabetes if HbA1c was <5.7%. *Lifestyle intervention* is recommended (metformin prevention considered for select high risk populations).[54] Diabetes requires fasting blood glucose ≥126 mg/dL or HbA1c ≥6.5%. If not-fasting, random blood glucose ≥200 mg/dL would be concerning for diabetes if HbA1c was <6.5%. In addition to lifestyle modifications, *metformin* is typically started with additional agents as indicated. We will ascertain the prevalence of pre-diabetes and diabetes; both may be eligible for Aim 2.

Undertreatment defined for each as:

- While any participant who was previously undiagnosed is also technically undertreated, we will reserve this definition to those who previously or currently reported to CCSS as being diagnosed with hypertension, dyslipidemia, or diabetes (either managed by lifestyle modifications or medication therapy), if their home-sampled value falls outside the recommended therapeutic range: blood pressure ≥130/80 mmHg, LDL ≥160 mg/dL, triglyceride ≥150 mg/dL (if fasting <10 hours: ≥200 mg/dL), or HbA1c ≥7.0% (different than the HbA1c diabetes diagnosis threshold[65]).

3.3.2 **Primary Aim 2 Endpoint:** Probability of intervention subjects having an undertreated CV condition compared with the control group at 1-year follow-up.

3.3.3 **Primary Aim 3 Endpoint:** Barriers among survivors and their primary healthcare providers that contribute to undertreatment of hypertension, dyslipidemia, and diabetes.

3.4 Estimated Accrual

The proposal would seek to enroll up to 800 participants to answer Primary Aim 1. If 60% of those participants are found to have an abnormal CV condition, up to 480 would then be eligible to participate in the randomized controlled trial as part of Primary Aims 2 and 3 (n=240 in each study arm).

3.5 Sponsor/Funding Source

The study is funded by the National Institutes of Health (National Cancer Institute).

4.0 SAFETY CONSIDERATIONS

We believe this protocol/study will be minimal risk. The proposed home visits and biosample and data collection pose minimal risk to participants. Anthropometric measurements are all non-invasive. There would be a blood draw (thus potential for brief mild pain) associated with any home visit (1 to 2 over the

course of the study for any given participant). The study team will ensure that the amount drawn at any visit is safe from a blood volume standpoint. It is possible, but unlikely, that some survey questions may cause participants to feel uncomfortable. All instruments proposed have been previously used in other research. For participants selected to participate in the randomized intervention trial, it is possible that receipt of clinical lab results may cause some participants to feel uncomfortable or anxious. Similarly, receipt of a SCP with an individual's predicted cardiovascular risk may cause anxiety or discomfort. We will be monitoring self-reported anxiety and depression as part of our study. Prior CCSS studies have not shown that receipt of such survivorship care plans to be a source of significant anxiety.[43] We feel that providing such information, even when unexpected, may be important and have important future health consequences, as the research data to be provided to participants are all results with clinical interpretation and significance. For participants on the intervention arm, they will get a phone or web video session by which any results are given and explained to them by a survivorship trained clinician, approximating a normal clinical exchange. Participants on the control arm will only get a letter summarizing clinically relevant results, and will be encouraged to discuss any abnormal results with their primary healthcare provider. The study has in place, a process by which critical test results will be reported to the study team in real time, in which case, the PI or his designee, can notify the participant more quickly to seek medical follow-up. This process is similar to and adapted from that used by the CDC-sponsored NHANES for their in-person mobile assessments. For participants (either intervention or delayed control) with clinically actionable results but who have no current healthcare provider, the study team has access to resources to aid participants in finding providers in their area who have expertise in treating survivors of childhood cancer. This will be clearly explained on their results letter.

5.0 SUBJECT ELIGIBLITY

5.1 Inclusion Criteria

For Primary Aim 1:

5.1.1 CCSS participant who is age ≥18 years at time of initial consent.

5.1.2 High CV risk status based on CCSS risk prediction models for cardiomyopathy and ischemic heart disease.

5.1.3 Living in the U.S., within 50 miles of a designated exam service provider (e.g., EMSI) based on CCSS's available contact information at the time of approach.

5.1.4 Able to read, write, and speak English.

5.1.5 Ability to understand and the willingness to provide informed consent.

For Primary Aim 2, in addition to the satisfying the above criteria, participants must also meet the following criteria:

5.1.6 Abnormal CV condition identified on home visit. Defined as having at least one of the following:
- Average blood pressure ≥130/80 mmHg (≥130/80 if existing hypertension diagnosis)
- LDL ≥160 mg/dL
- Triglyceride ≥150 mg/dL (if ≥10 hours fast) or ≥200 mg/dL (if <10 hours fast)
- If not known to be diabetic: Glucose ≥100 mg/dL (if ≥8 hours fast) or ≥140 mg/dL (if <8 hours fast)
- HbA1c ≥5.7% (if not known to be diabetic), HbA1c ≥7% (if known diabetic).

5.1.7 Be free of any known (self-reported) ischemic heart disease or cardiomyopathy.

5.1.8 Have access to a telephone or computer to receive a phone or web video counseling/intervention session at baseline and at 4 months.

5.2 Exclusion Criteria, applicable to all participants

5.2.1 Individuals with known cardiomyopathy or ischemic heart disease based on prior CCSS surveys are excluded. While not likely to be common, participants who *newly* report in our study's baseline survey that they have cardiomyopathy or ischemic heart disease can have a home visit completed but will then be done with the study regardless of their home visit results.

5.2.2 Not currently known to be pregnant; individuals known to be pregnant and otherwise eligible for the study can be enrolled once no longer known to be pregnant. Participants who report being pregnant AFTER randomization can remain in the study.

5.2.3 Individuals receiving active cancer treatment. Participants who report starting active cancer treatment AFTER randomization can remain in the study.

6.0 SUBJECT RECRUITMENT & REGISTRATION

6.1 Recruitment

A list of eligible patients for Aim 1 will be generated using existing CCSS data. Staff at the CCSS Data Coordinating Center at St. Jude will be responsible for sending out approach packets to these patients (introductory letter, Consent, Healthcare Provider Form, HIPAA, study questionnaire, and upfront honorarium). We will also have the Consent/ Healthcare Provider Form/ HIPAA Form and the study questionnaire available online. For non-responders, St. Jude interviewers will follow-up with phone calls using their standard process. *As part of St. Jude's standard CCSS process for other CCSS activities, participants will have the option of indicating consent by signing the paper form, signing an online consent, or by providing verbal consent to a CCSS St. Jude interviewer.* Similarly, questionnaires can also be completed by interview.

With our 12/2017 protocol modification, we will also pilot alternative approach packets to see if smaller packets that request less of participants upfront can improve accrual. Specifically, in addition to the existing approach process, we will pilot a 2nd process by which we exclude the printed study questionnaire from the approach packet (an option to complete this on-line will still be mentioned in the cover letter). Participants who consent in this fashion but who do not complete the on-line questionnaire will then be sent a paper version. A 3rd process will also be piloted which will feature a tiered consent process by which the initial consent will just be to complete the initial home visit without consenting to the subsequent randomized intervention trial. For those participants who consent in this fashion, after they complete the initial home visit and are found to be eligible for the randomized intervention trial, they will then receive the second consent form that describes the trial. If one of these alternative approach processes results in a substantially higher enrollment rate, that process will then be adopted as our standard approach.

Contact information for interested participants will then be forwarded to a designated exam service provider (e.g., EMSI) who may then directly communicate and schedule a home visit with the participant. As part of the home visit, for those participants who did not provide a paper-signed copy of their HIPAA authorization / Medical Record Release form (since we are unsure electronic or verbal signatures will be acceptable for primary care providers' offices), staff will also ask participants for their willingness to sign a HIPAA authorization and Medical Record Release (to enable the study to obtain their primary care provider's medical records).

Based on the study's eligibility criteria, we do not believe patients who are approached will be subsequently found to be ineligible. It is possible (although unlikely) that we may identify someone who has died since their last CCSS contact. CCSS staff at St. Jude are trained to deal with this situation and CCSS records will be updated accordingly. Also unlikely but possible, an otherwise eligible participant may have moved and may now live in at an address outside the exam service provider area. Should this occur, the study will send the participant a thank you/acknowledgment letter indicating that they will not be able to participate this time (**Appendix**).

6.2 Registration

Information on consented patients will then also be forwarded to the Fred Hutch study coordinator and entered into a study specific tracking database. A copy of the signed consent form and HIPAA authorization and Medical Record Release will also be sent to the Fred Hutch study coordinator.

7.0 TREATMENT PLAN

7.1 Treatment Plan Overview

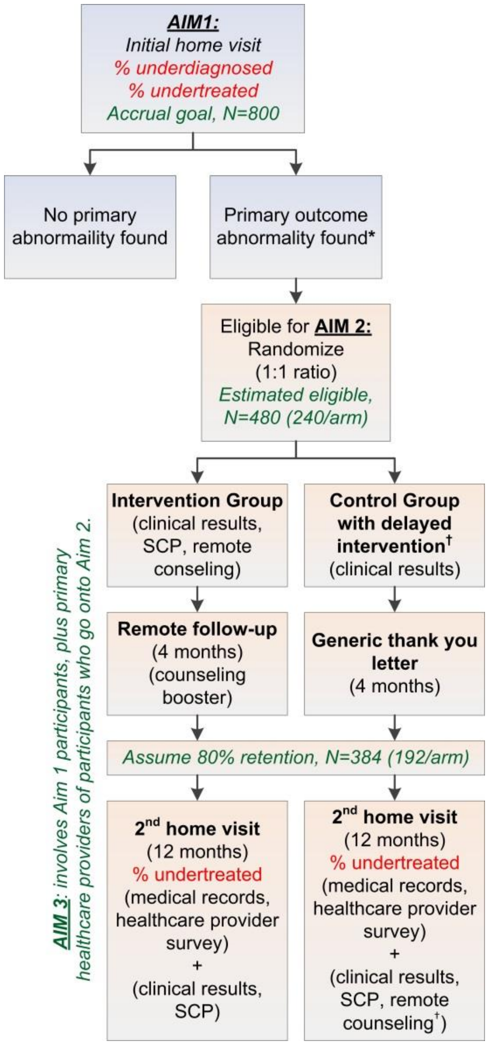

* ≥1 of the following: blood pressure ≥130/80 mmHg (≥130/80 if existing hypertension diagnosis); LDL ≥160 mg/dL; triglyceride ≥150 mg/dL (fasting) or ≥200 mg/dL (non-fasting); if not known to be diabetic, glucose ≥100 mg/dL (fasting) or ≥140 mg/dL (non-fasting) or HbA1c ≥5.7% (if not previously diabetic), HbA1c ≥7% (if known diabetic). †Controls can receive the intervention *after* the 2nd home visit & survey completion.

7.2 Assignment of Study Group

Among those who meet eligibility criteria for primary aim 1 but not primary aim 2, participation will end after the 1st home visit. Similarly, if we adopt a tiered consent process, those who could be eligible for aim 2 but who do not provide consent to the randomized intervention portion of the study, their participation will end after the 1st home visit.

Among participants who meet eligibility for primary aim 2, there this will be a computer randomization on a 1:1 basis to intervention: control assignment, with the control group eligible to receive the intervention on a delayed basis (following the 2nd home visit). The study's goal will be to review in-person collected test results in real-time (within 1-2 days of being received at St. Jude) and determine eligibility with randomization assignment the same day. Given the intervention, it cannot be blinded to

either participant or study clinician. Staff who perform the home visits and the study biostatisticians will be blinded to the study group assignment.

7.3 Intervention Group

The intervention is a personalized clinician-led SCP-focused self-management counseling session that is generalizable to a real clinical setting. A printed SCP with personalized health history, recommendations, and clinically meaningful results from the initial home visit (i.e., average blood pressure, lipid profile, diabetes screening, BMI) will first be mailed to survivors on the intervention arm. We will then schedule participants for a remote counseling session via telephone or HIPAA-compliant web video if the participant prefers. The 1st session with a study clinician will ideally be ≤30 minutes to review the SCP and make mutually agreed upon goals and an action plan (see **Appendix**). We will ask scripted questions designed to address understanding of the SCP, elicit intentions to act on the SCP information, and elicit potential barriers and their solutions related to any planned actions. After the session, the personalized action plan will be mailed to the participant.

Approximately 4-months after the initial counseling session, participants will schedule a 2nd session by telephone, or HIPAA-compliant web video if the participant prefers, with the study clinician to follow-up on the action plan, address any barriers to the plan and mutually agree on a revised plan if needed (ideally ≤15 min). The clinician will also rate the survivor's completion of the action plan from 0 (none of the plan completed and no apparent intention to complete the plan) to 10 (action plan complete; see **Appendix**).

For the both the baseline and 4-month follow-up sessions, the duration of the sessions will be recorded to determine the feasibility of delivering the intervention within the time allotted (≤30 and ≤15 minutes, respectively). The sessions also will be audiotaped for process evaluation of content and fidelity of the clinician adherence to the intervention manual. This is further described in **Section 8**.

7.4 Delayed Intervention Control

Survivors in the control arm will receive a copy of clinically meaningful baseline home visit results, with a general recommendation to seek medical follow-up in instances where any of those values are abnormal since we consider it unethical to withhold such information. A copy also will be sent to their designated healthcare provider. *As such, the control group's health may be improved simply by study participation.* At 4-months, participants will receive a generic thank you letter by mail for participating. This will include a reminder of the 2nd home visit, and to enhance control group retention, control participants will be told that they can receive the study intervention (minus the 4-month booster session) after completing the 2nd home visit and any associated surveys. This will include reviewing an SCP and self-management strategies with the study clinician, but we will avoid using those exact terms to minimize control group contamination.

7.5 Interaction with Primary Healthcare Providers

All participants, at time of consent, will be asked to list their current and past (within the last 2 years) primary healthcare provider(s). Current designated providers will receive a copy of all materials sent to study participants. Thus, healthcare providers of participants randomized to the intervention arm will be mailed a print copy of the SCP with home visit results and the survivors' personalized action plan. We will directly send the SCP to providers because studies have shown that reliance on survivors alone to disseminate a copy of their SCP to their provider is problematic.[46,55] An analysis of existing CCSS data showed that receipt of a print media intervention and fostering increased survivor-physician dialogue was associated with increasing adherence to screening.[26] Providers can contact the research team with

any questions regarding the SCP and action plan contents.

- Following the 1st home visit, healthcare providers designated by participants randomized to the control arm will be mailed a print copy of the home visit results only.

- Following the 2nd home visit, healthcare providers of participants on both study arms will be surveyed with regards to their self-efficacy (including self-reported knowledge) towards providing care for childhood cancer survivors. The survey will be accompanied by a cover letter, copies of clinical results (both groups), a SCP (intervention group only), and the patient's signed HIPAA authorization and Medical Record Release (if available), requesting records from the past 3 years. If the budget permits, the study will provide an upfront honorarium to providers (not exceeding $20). Non-responding providers will receive a 2nd survey packet (inclusive of clinical results, SCP if applicable, and the signed HIPAA authorization and Medical Record Release if available) approximately a month later with an alternative cover letter. No honorarium will accompany the 2nd packet. This will be faxed to the provider's office if a fax number is available, and/or mailed. We will repeat this process two more times if no response is received after another month.

For participants (either intervention or delayed control arm) with clinically actionable results but who have no current healthcare provider, the study team has access to resources to aid participants in finding providers in their area who have expertise in treating survivors of childhood cancer. This will be clearly explained on their results letter and by phone or web-video.

7.6 Duration of Therapy and Follow-Up

All study participants will be followed through their 2nd home visit, approximately 1-year after their 1st home visit unless they withdraw consent from the study prior. All participants following the 2nd home visit will receive a copy of their clinically relevant results embedded in a SCP. As mentioned above, those on the control arm can then have the option of having a 30 minute counseling session to review those results and the SCP with the study clinician.

8.0 STUDY PROCEDURES

An overview of study procedures can be found in **Table 3**.

TABLE 3. *Summary of required observations.*

	**Initial home visit**	**Baseline counseling session**	**4-month booster**	**2nd home visit**
Intervention group	Questionnaires*<br>Anthropometrics<br>Blood draw	Goals/Action Plan<br>Call duration<br>Call fidelity	Rating of Action Plan completion	Questionnaires*<br>Anthropometrics<br>Blood draw
Control group	Same as intervention group	-	-	Same as intervention group
Primary healthcare provider(s)	-	-	-	Provider survey<br>Clinical records
Time window to complete	<2 mo of enrollment (max: <6 mo)	<2mo of home visit (max: <3 mo)	3-5 mo from baseline	11-15 mo from 1st home visit (no max))

*Participants will be asked to complete the questionnaires prior to the scheduling of their home visits.

8.1 Baseline Questionnaire

This will include items on: 1) past medical history, 2) current health and medication adherence, 3) lifestyle habits, and 4) attitudes towards healthcare and current mood (**Table 4; Appendix**).

TABLE 4. *Components of baseline questionnaire, exclusive of questions on past medical history and lifestyle habits.*

***Health-related self-efficacy**[56]	10-item (4-point Likert scale) survey (will be reduced to 5-items) that measures perceived ability to set-goals, cope and recover from setbacks. Results have been found to be a strong predictor of subsequent health behavior change.[96]
***PROMIS anxiety & depression**[57,58]	These domains (adult 4-item survey; all 5-point Likert scale) are of secondary interest. Prior research via a longer instrument (Profile of Mood States) did not show SCPs associated with mood changes or anxiety.[50,85]
***Multidimensional Health Locus of Control [MHLC]**[59]	18-item survey (6-point Likert scale) will classify participants with regards to their attitudes towards healthcare screening into "worried", "collaborative" and "self-controlling" typologies, which have been associated with differential likelihoods of obtaining recommended screening in retrospective CCSS analyses.[78]
***Medication Adherence Scale (MMAS-4)**[60]	Well-established 4-item (0=high, 1-2=medium, 3-4=low adherence) instrument initially used to study hypertension, but since validated for other diseases.[100] Will be administered separately for blood pressure, lipid, and diabetes medications. Secondary analyses will examine adherence by medication burden.

8.2 First Home Visit

This will include:

- Signature on HIPAA authorization and Medical Record Release form (optional).
- Standing height: in centimeters, to the nearest 0.1 cm.
- Weight: in kilograms, to the nearest 0.1 kg.
- Blood pressure at rest: 3 times; each at least 3 minutes apart.
- Waist circumference: in centimeters, to the nearest 0.1 cm; just above the uppermost lateral border of the right ilium [hip bone]; at the end of a normal expiration.
- Blood draw (no fasting required): lipid profile, blood glucose, hemoglobin A1c, insulin, and samples for ancillary biological studies (**Table 5**).

TABLE 5. *Overview of blood samples, draw order, vial type, volume.*

Draw order / Analyte	Vial type	Volume
1) Lipid profile, glucose, insulin level*	Heparin plasma separator tube (green top)	3.0 mL
2) Hemoglobin A1c*	EDTA tube (lavender top)	1.0 mL
3) Protein & DNA-based assays	EDTA tube (lavender top)	7.5 mL
4) General chemistries	Serum separator tube (red/gray top)	7.5 mL
5) Proteomics, metabolomics	EDTA separator tube (pearl)	7.5 mL
6) RNA for gene expression	PAXgene tube	2.5 mL

*These also will be drawn at the 2nd home visit; remaining tubes would NOT be drawn at the 2nd home visit unless they could not be successfully drawn at the first visit. 2nd home visit only: blood spot offered for failed venous draws.

With our 7/2020 protocol modification, in response to the unexpected closure of the study's original exam service provider (EMSI), replacement service providers may include those that do not provide home-based services but rather fixed laboratory / service centers that the participant travels to in order to obtain the specified research blood draw. In these instances, only tubes 1-2 (**Table 5**) will be drawn and processed on-site (i.e., tubes 3-6 will not be drawn).

Rarely, in past experiences with our pilot data and similar CCSS home visit-based studies, exam service staff will be unsuccessful in obtaining blood given technical issues. In this situation, the participant will be offered the choice of having the procedure be rescheduled to another date. *If the participant refuses and the primary study endpoints (lipid profile, blood glucose, and hemoglobin A1c) cannot be collected, then the patient will not be eligible to continue on the study.* Any partial results will still be sent back to the participant and their designated primary care provider.

8.3 Baseline Counseling Session (Intervention Group)

A printed SCP with personalized health history, recommendations, and clinically meaningful results from the home visit (i.e., average blood pressure, lipid profile, diabetes screening, BMI) will first be sent to survivors on the intervention arm. Interpretation of these results is as defined in **Section 3.3.1.** We will then schedule participants for a remote counseling session by phone or web video, per participant choice). This will be scheduled as soon as possible following the initial home visit with a goal of having this being completed within 2 months of the home visit. Sessions completed outside of the 2-month window will be flagged (but remain eligible for analysis). The following outcomes will be measured at this time point:

- Creation of a personalized goals and action plan **(Appendix**).

- Counseling session completion rates, with goal of obtaining ≥85% completion within 2 months of the initial home visit.
- Call duration, with goal of achieving ≥85% being ≤30 minutes in duration.
- Study clinician fidelity to the intervention. Fidelity will be scored using standard methods across 8 levels with the 8th a global rating (**Appendix**). Trainers will review audiotapes of sessions, with fidelity ratings and feedback to the clinician until the mean of 4 consecutive global ratings are ≥3.5 (scale: 0 [poor] to 5 [very good]). Once certified, trainers will review audio recordings and complete fidelity ratings for 15% of sessions, more if fidelity dips below a global rating of 3 (satisfactory) for 3 of 10 sessions.

It is possible that some randomized participants will fail to schedule a session despite multiple contact attempts. *We will cease scheduling attempts if no session has been scheduled by 3 months after the initial home visit.* These participants will not be eligible for the 4 month booster session (discussed below). However, we will still attempt to re-assess their health status at the 1-year time point with a 2nd questionnaire and home visit. ***If a participant has to cancel and reschedule, the rescheduled date can fall outside the 3 month deadline and we will attempt to do the booster session within the target time range still.***

8.4 Baseline Return of Results (Control Group)

Survivors in the control arm will receive a copy of clinically meaningful baseline home visit results, with a general recommendation to seek medical follow-up in instances where any of those values are abnormal since we consider it unethical to withhold such information. Interpretation of these results is as defined in **Section 3.3.1.** A copy also will be sent to their designated healthcare provider. These will be issued within one month of the home visit.

8.5 Handling of Critical Results

Staff will be asked to ship out blood samples within 24 hours (could be slightly longer on weekends) of collection, and data forms will be faxed, both to the CCSS data coordination center at St. Jude. Once received, labs will be processed in real-time (i.e., not batched) by the CLIA-certified lab at St. Jude. Copies of data forms and any examiner shipping manifest will be forwarded to the study team at FHCRC. In the event that a critical test result is found (see **Table 6**), the PI or his designee, will attempt to notify the participant more quickly by phone to seek medical follow-up (rather than waiting for a letter to be generated and mailed to the participant). This process is similar to and adapted from that used by the CDC-sponsored NHANES for their in-person mobile assessments.

TABLE 6. *Classification of test results, as discussed in Section 8.5.*

	Goal	Borderline	Abnormal	Critical
Systolic blood pressure (mmHg)*	<120	120-129 systolic	130-179	≥180
Diastolic blood pressure (mmHg)	<80		80-119	≥120
LDL cholesterol (mg/dL)	<130	130-159	≥160	-
Triglyceride (mg/dL), if fasted ≥10 hrs	<150	-	150-749	≥750
Triglyceride (mg/dL), if not fasting	<200	-	200-749	≥750
Glucose (mg/dL), if fasted ≥8 hrs	<100	100-125	126-299	≥300† or ≤50‡
Glucose (mg/dL), if not fasting	<140	140-199	200-299	≥300† or ≤50‡
Hemoglobin A1c (%)	<5.7	5.7-6.4	≥6.5	-

*We will drop the highest of 3 systolic measurements, and average the remaining 2 for systolic & diastolic values. †If not previously known to have a diagnosis of diabetes. ‡If known diabetic only, as this would be concerning for therapy-related hypoglycemia.

8.6 Four-month Booster Session

Among the intervention group, the study will attempt to schedule the participant for a brief 15-minute check-in by phone or web-video 4-months (±1 month) after the initial counseling session. For intervention subjects who have such booster sessions outside the 3-5 month window, their data will be flagged but would still remain eligible for analysis. The following outcomes will be captured at this time point:

- Study clinician rating of participants' action plan completion, ranging from 0 (none of the plan completed and no apparent intention to complete the plan) to 10 (action plan complete; see **Appendix**).
- Similar to the baseline session, we will cease trying to schedule this booster session if no session has been scheduled by 5 months after the initial counseling session. We will still attempt to re-assess their health status at the 1-year time point with a 2nd questionnaire and home visit. *If a participant has to cancel and reschedule, the rescheduled date can fall outside the 5 month deadline.*

Among the control group, participants will receive a generic thank you letter by mail for participating. No outcomes will be measured in this group at this time point.

8.7 Second Questionnaire and Home Visit

The 2nd questionnaire and home visit should take place approximately 1-year after the initial questionnaire and home visit. The study will send participants an abridged version of the baseline questionnaire (by mail, with phone/email follow-up as necessary) with a reminder that a 2nd home visit is also due soon. After the participants complete the follow-up questionnaire, the study will work with the exam service provider to contact the participant to arrange for the 2nd home visit. The study will attempt to have all 2nd home visits occur within 3 months of the 1-year anniversary, and visits that occur outside that 3-month window will be flagged (but remain eligible for analysis). The following outcomes will be measured at this time point:

- Same physical measurements as before: standing height, weight, blood pressure at rest (3 times), and waist circumference.
- Blood draw (no fasting required): lipid profile, blood glucose, hemoglobin A1c, and insulin (**Table 5**; other assays will not be drawn unless they could not be drawn at the first home visit).
- If the exam service provider is unsuccessful in obtaining blood due to a technical issue, or the participant refuses the venous blood draw at the second visit, we will offer the participant the option of a finger poke to obtain a blood spot sample (i.e., dried blood spot, DBS). Blood spots will only be offered as a back-up option at the second visit.
- Handling of any critical results will be conducted similarly as per the baseline home visit (see **Section 8.5** above). In contrast to venous samples, DBS-based measurements of the lipid profile, glucose, insulin, and hemoglobin A1c will <u>not</u> be released back to participants or their providers. This is because such measurements are not performed in a CLIA-certified setting and are currently designed for epidemiologic research only (and not for clinical care), and also will be batched (in contrast to venous samples which are processed in near real-time).

Should home visits be completely declined or impossible to conduct (e.g., due to COVID-19 or other public health issues where social distancing is encouraged/mandated), the protocol will permit

substitution of a completely remote assessment procedure for the follow-up visit. This would necessarily be slightly modified from the exam service provider-obtained home visit in the following ways:

- Physical measurements will only include: weight and blood pressure at rest (3 times). These will be collected using a scale and blood pressure cuff that the study can provide to study participants. If participants already have a digital scale at home, they can use their own scale in place of a scale the study provides. Blood pressure cuff and scale can be kept by participants and do not need to be returned to the study team. These results can be returned to participant and their primary healthcare provider, but would be clearly noted as participant-collected.
- Blood draw will be replaced by the DBS method specified above, with the same caveat that DBS results cannot be returned to participants.

8.8 Primary healthcare provider measurements

Following the 2nd home visit, accompanying the mailing of a copy of participant materials (intervention group: clinical results and SCP; control group: clinical results only), all designated providers will be asked to answer questions adapted from the NCI/ACS SPARCCS (see **Appendix**).[61]

We will also request outpatient clinical records (clinician notes, medication lists, laboratory results) spanning the study period (and up to 2 years prior to the 1st home visit) from all participants' primary healthcare providers. We will abstract records for: 1) documentation of the participant's prior cancer history and CV risk status in relation to past cancer exposures; 2) any reference to a SCP or long-term follow-up guideline; 3) any CV screening planned or undertaken [coded separately]; 4) presence of any of the three target CV risk factors as a diagnosis/problem; and 5) interventions to address any CV risk factor [coding lifestyle or drug prescription separately]. Medications will be reviewed to examine whether, among those undertreated at the 1st home visit, there is subsequent treatment intensification (e.g., going from lifestyle prescription alone to adding medication; from lower to higher drug doses; from single to multiple drugs; **Table 7**), and whether treatment intensity differs across study arms after the intervention.

***TABLE 7.** Examples of CV medications & dose categories (mg)*

**Medication (class)**	**Low**	**Medium**	**High**
Hypertension			
Lisinopril (ACE-inhibitor)	≤10	11-30	>30
Amlodipine (calcium channel blocker)	≤2.5	2.6-9.9	≥10
High cholesterol			
Simvastatin (HMG CoA reductase inhibitor)	<20	20-39	≥40
Diabetes			
Glipizide (sulfonylurea)	<10	10-19	≥20
Insulin		Any dose	

Description of how the study will engage with primary healthcare providers is otherwise described in **Section 7.5.**

8.9 Materials returned to participants and primary healthcare providers

Information the study will send (by mail) back to study participants and their designated current primary healthcare provider are summarized in **Table 8**.

***TABLE 8.** Information the study will mail back to study participants and their current primary healthcare providers.*

	**After the initial home visit / baseline counseling session**	**After the 4-month booster**	**After the 2nd home visit**

Intervention group (participant & provider)	Clinical results<br>Survivorship care plan<br>Action Plan	Updated Action Plan	Clinical results<br>Survivorship care plan
Control group (participant & provider)	Clinical results	-	Clinical results<br>Survivorship care plan*
*Mailing of the care plan would not occur until AFTER the control group's designated healthcare providers are surveyed.			

8.10 Remote blood pressure monitoring pilot

Following completion of the intervention, we will also approach participants regarding their willingness to perform home blood pressure monitoring (HBPM) via mHealth (i.e., Bluetooth-enabled) devices. The rationale for this is that compared with our standard measurement approach, home monitoring is likely to be more accurate, both in ruling out "white coat" hypertension as well as ruling in "masked" hypertension.[50] This pilot would seek to enroll 40 participants who have just completed their 2nd home visit (or 1st home visit if all values were normal and they were otherwise not eligible to participate in the randomized controlled trial) and ask them to collect home blood pressures 3 times a week for 4 weeks. A Bluetooth-enabled blood pressure monitor would be provided free of cost to study participants. Participants would be requested to download the blood pressure manufacturer's mHealth application (app) which will collect measured blood pressure values as well as a 2nd study-specific mHealth app (Eureka, UC San Francisco), which is designed to facilitate mHealth research and transmit research-specific reminders (e.g., phone notifications or texts) and instructions to participants, as well as provide investigators the ability to access the blood pressure values collected on the monitors.

The primary goals of this pilot would be to determine the feasibility of HBPM, including participation rate and the proportion of participants who transmit >75% of requested data (>9 measurements in one month). Secondary goals would be to determine the proportion of individuals who may have "white coat" hypertension (i.e., in-person collected values are systolic ≥130 or diastolic ≥80 mmHg but HBPM average is lower) or "masked" hypertension (i.e., in-personcollected values are systolic <130 and diastolic <80 mmHg, but average HBPM is greater). Data from this pilot will be used to inform the feasibility of a future follow-up study that involves using HBPM as part of the study assessment and intervention.

Participants would be approached as part of their final home visit results mailing, and those interested would be instructed to download the Eureka app onto their smartphone and provide informed consent on that app. The study team would follow-up by phone (including text messaging) and/or email among non-responders to determine interest. Participants would be eligible for an additional incentive upon completing this 1-month pilot (choice of either keeping their home blood pressure monitor or a $50 gift card). As all blood pressure measurements will be visible to participants on both the monitor as well as the manufacturer's app on their phones, they will be encouraged to share any values with their healthcare provider if they have concerns.

Specific eligibility for this follow-up pilot study would include:

- CHIIP study participant who has completed their 2nd home visit (or 1st home visit if all values were normal and they were otherwise not eligible to participate in the randomized controlled trial);
- Has access to an internet-connected smart phone capable of downloading apps from either Apple or Android-based "app stores";

- Consents to this follow-up study, which includes being willing to download both the blood pressure monitor manufacturer app as well as research Eureka app, and being willing to receive study-specific reminders (phone notifications and/or text reminders);
- Arm circumference is within the parameters of the home blood pressure monitor manufacturer specifications.

9.0 OFF STUDY CRITERIA

This would occur if:

- A participant withdrew consent for any further data submission, or
- The participant was unable to complete the initial home visit procedures (e.g., unsuccessful blood draw), or
- Once a participant has completed all study assessments.

10.0 ADVERSE EVENTS

10.1 Expected Adverse Events

The CHIIP study is considered a low-risk educational and behavioral intervention study. The proposed home visits and biosample and data collection itself pose minimal risk to participants. Anthropometric measurements are all non-invasive. There would be a blood draw (thus potential for brief mild pain) associated with any home visit (1 to 2 over the course of the study for any given participant). The study team will ensure that the amount drawn at any visit is safe from a blood volume standpoint. It is possible, but unlikely, that some survey questions may cause participants to feel uncomfortable. All instruments proposed have been previously used in other research.

For participants selected to participate in the randomized intervention trial, it is possible that receipt of clinical lab results may cause some participants to feel uncomfortable or anxious. Similarly, receipt of a SCP with an individual's predicted cardiovascular risk may cause anxiety or discomfort. We will be monitoring self-reported anxiety and depression as part of our study. Prior CCSS studies have not shown that receipt of such survivorship care plans to be a source of significant anxiety.[43] We feel that providing such information, even when unexpected, may be important and have important future health consequences, as the research data to be provided to participants are all results with clinical interpretation and significance. For participants on the intervention arm, they will get a phone session or web video by which any results are given and explained to them by a survivorship trained clinician, approximating a normal clinical exchange. Participants on the control arm will only get a letter summarizing clinically relevant results, and will be encouraged to discuss any abnormal results with their primary healthcare provider. The study has in place, a process by which critical test results will be reported to the study team in real time, in which case, the PI or his designee, can notify the participant more quickly to seek medical follow-up. This process is similar to and adapted from that used by the CDC-sponsored NHANES for their in-person mobile assessments. For participants (either intervention or delayed control) with clinically actionable results but who have no current healthcare provider, the study team has access to resources to aid participants in finding providers in their area who have expertise in treating survivors of childhood cancer. This will be clearly explained on their results letter.

Otherwise, the only other anticipated risks would be related to participant privacy. The PI and study team will have clear and standard procedures to manage and protect individually identifiable private information. These include: appropriate human subjects research training for all staff with potential access to private information; technological safeguards such as use of encrypted data transmission between sites, secure password protection on computer servers; removal of identifiers at the earliest possible time and using separate unique study IDs instead; keeping any cross-walk files separate from the main analytic dataset(s); and avoiding showing individual data in any presentation or publication.

10.2 Monitoring and Recordings Adverse Events

As the CHIIP study is considered a low-risk educational and behavioral intervention, the FHCRC Data Safety Monitoring Committee, during the study's grant application, made an initial determination that overall data and safety monitoring can be overseen by the PI and the FHCRC IRB. All adverse events (expected or unexpected) will be reported to the FHCRC and St. Jude IRBs in accordance with the respective IRBs' policies.

11.0 DATA AND SAFETY MONITORING PLAN

The study will use the FHCRC Data and Safety Monitoring Committee (DSMC). The DSMC will review the summary of all adverse events collected as part of a protocol's data safety monitoring plan and reported by the PI at time of continuing IRB renewal, any expedited reports made to the IRB, and may refer protocols to the IRB for evaluation when modifications might be required to mitigate risks. The DSMC may also suspend or close the study based on review of the above information. The DSMC will review the accrual, patient safety, and other data at pre-specified intervals (at least annually), and make any determinations regarding interim analyses and stopping rules. On the basis of its review, the DSMC will provide a study with full approval (accrual continues, no changes), conditional approval (accrual continues but protocol changes required), suspension (accrual is stopped until protocol changes have been made but previously enrolled patients may continue the intervention), or closure (accrual is stopped and all interventions must stop).

12.0 DATA MANAGEMENT/CONFIDENTIALITY

The investigators will ensure that data collected conform to all established guidelines. Each participant has a pre-assigned CCSS ID number that will be used to protect subject confidentiality. Subjects will not be referred to by this number, by name, or by any other individual identifier in any publication or external presentation. Subject research files will be stored in a secure place (or database). Access is restricted to authorized personnel, with supervision by the PIs at FHCRC (Chow) and St. Jude (Armstrong). An existing data transfer/sharing agreement between FHCRC and St. Jude covers the exchange and sharing of existing and newly collated CCSS data. Data will not be shared with any other entity or outside investigators (external to those approved by CCSS) without approval by the relevant IRBs.

13.0 STATISTICAL CONSIDERATIONS

13.1 Aim 1 Analytic Plan

<u>Hypothesis</u>: Approximately 60% of participants will have an abnormal CV risk-factor finding that meets clinical intervention thresholds, and among those with abnormalities, 40% will be undertreated and 80% newly diagnosed.

<u>Primary analyses</u>: We will calculate the prevalence of hypertension, dyslipidemia, and diabetes by self-report and by physiologic measurement. We will then calculate the proportions (with exact 95% confidence intervals) of participants who may be underdiagnosed as well as the proportions undertreated (like **Figure 3**). We will calculate the prevalence of directly measured pre-diabetes separately. **<u>Secondary analyses</u>:** We will examine whether rates differ by: sex; current age; time since cancer diagnosis; obese (BMI ≥30 kg/m^2) vs. not; health insurance vs. not; recent history of being seen in a dedicated long-term follow-up clinic for survivors vs. not; and lifestyle factors (smoking, low fruit/vegetable intake, low physical activity).

<u>Sample size considerations:</u> Based on prevalence estimates from CCSS surveys and our pilot (**Table 2, Figure 3**), we expect ~60% of high CV risk participants (n~480) to have an abnormal CV risk factor measurement; prevalence of individual risk factors ranging 20-40% (less when stratified by underdiagnosis/undertreatment status). With an overall sample n=800, we expect reasonable precision for our primary prevalence estimates (**Table 9**). Power to detect differences <10% in secondary analyses may be more limited.

TABLE 9. *Estimated power for Aim 1 analyses.*

**Prevalence±95% CI**	**Minimum detectable proportions***	
**N=800**	**N=320:480**	**N=200:200**
5% ± 1.5%	5% vs. 1/11%	5% vs. 0/14%
20% ± 2.8%	20% vs. 12/29%	20% vs. 10/33%
50% ± 3.5%	50% vs. 40/60%	50% vs. 36/64%

*****2-sample Fisher's exact test, 80% power, α=0.05.

<u>Potential pitfalls / solutions:</u> Our EMSI pilot achieved 60% enrollment. Funding from this proposal will enhance recruitment rates by providing an upfront (rather than conditional) participation incentive, which has been associated with higher rates of study participation.[62,63] We will closely examine the demographic characteristics, past cancer treatment exposures, and self-reported burden of CV and other chronic conditions of survivors who are approached vs. not approached, and who ultimately participate vs. not. If differences are found, we will apply inverse probability weighting as a way to assess the sensitivity of our estimates.

13.2 Aim 2 Analytic Plan

<u>Hypothesis</u>: survivors randomized to the intervention will have a lower probability of having an undertreated CV risk factor compared with the control group at 1-year follow-up.

<u>Primary analyses</u>: We will describe baseline characteristics (including mean/median values of our target CV parameters) and proportions of underdiagnosis and undertreatment of our 3 CV risk factors. Our primary analysis will focus on the difference in the probability of having a CV risk factor undertreated after the 2nd home visit across the 2 study arms. As in Aim 1, undertreatment of hypertension, dyslipidemia, and diabetes at the 2nd home visit will be defined as blood pressure ≥130/80 mmHg, LDL ≥160 or triglyceride ≥150 mg/dL (if fasting <10 hours: ≥200 mg/dL), or HbA1c ≥7%, respectively. Survivors with <u>pre-diabetes at baseline</u> will be undertreated at the 2nd home visit if fasting glucose ≥100 mg/dL (non-fasting ≥140 mg/dL) *or* HbA1c≥5.7%. As some survivors may contribute up to 3 outcomes (persistent hypertension, dyslipidemia, and diabetes/pre-diabetes), we will use generalized estimating equations (GEE), accounting for intra-individual correlation of the 3 outcomes, to estimate the overall intervention effect as a single parameter (3-element vector). If multiple survivors share the same primary healthcare provider (unlikely), we will apply random effects models in lieu of GEE given multiple

cluster types. Analyses will be conducted per intent-to-treat, and include all survivors with available end points.

Secondary analyses: In case survivors who were newly diagnosed (i.e., underdiagnosed) following their 1st home visit differ in their response to the intervention vs. those previously diagnosed but undertreated, we will examine estimates stratified by diagnosis status at the 1st home visit. For similar reasons, we also will examine estimates if pre-diabetes are excluded. In our primary model, we also will explore whether differences vary by sex, current age, time since cancer diagnosis, obese (BMI ≥30 kg/m^2) vs. not, health insurance vs. not, and recent history of being seen in a dedicated long-term follow-up clinic for survivors vs. not. We also will explore, among intervention participants, whether action plan scores from the 4-month session are associated with differential undertreatment rates (as a single parameter). Finally, we plan to also compare across the study arms, the measured CV risk factor values from the 2nd home visit separately: 1) average systolic pressure, 2) average diastolic pressure, 3) LDL, 4) triglyceride, 5) blood glucose, and 6) HbA1c. For this subanalysis we will use linear models, adjusting for the value at the 1st home visit as a covariate.

Sample size considerations: Assuming 60% of Aim 1 participants (n=800) meet Aim 2 eligibility with a subsequent conservative 20% drop-out rate, ~380 survivors (190/arm) would have complete data. The study would be 80% powered (α=0.05) to detect RRs ≤0.88 (i.e., ≥12% reduction in the intervention vs. control group) if the prevalence of having an undertreated CV risk factor among controls after the 2nd home visit was 90% (10% reduction from baseline). If controls had an unlikely 50% reduction, we can still detect RRs ≤0.71. Analyses stratified by initial underdiagnosis or undertreatment status (~75-150/arm) will be able to detect RRs ≤0.79 to ≤0.86, respectively. Systematic reviews have reported RRs 0.6-0.8 for strategies similar our proposed interventions in the general population.[64,65] For continuous outcomes (secondary analyses), we will be powered to detect change equaling ~30% of 1 standard deviation, which equates to 5 mmHg systolic blood pressure, 3 mmHg diastolic pressure, 10 mg/dL LDL, 30 mg/dL triglyceride, 10 mg/dL glucose, and 0.3% HbA1c. Differences within these ranges have been reported by others for interventions conducted in the general population.[66-68]

Potential pitfalls / solutions: We will closely monitor rates of abnormalities identified in Aim 1, as that influences the number of survivors eligible for Aim 2. As discussed earlier, we will oversample survivors known to have rarer target conditions (e.g., diabetes) such that the power to detect differences in Aim 2 is enhanced. Separately, given the nature of the intervention, participants and the study clinician cannot be blinded. However, exam staff will be blinded to randomization status.

13.3 Aim 3 Analytic Plan

Hypotheses: 1) at baseline, underdiagnosis & undertreatment are associated with lower knowledge, self-efficacy, and medication adherence (if applicable), as well as a "self-controlling" health attitude among survivors; 2) at 1-year follow-up, compared with the control arm, the intervention arm will be associated with improved knowledge, self-efficacy, and adherence rates among survivors, and improved knowledge and self-efficacy among healthcare providers.

Baseline survivor-specific factors: Using data from all Aim 1 participants, determine whether those underdiagnosed and undertreated at the time of the 1st home visit have lower knowledge (i.e., less accurate recall of prior anthracycline or chest radiotherapy exposures [chi-square test]) and lower health-related self-efficacy [t-test or Wilcoxon rank-sum] compared with those not affected. Similarly, among those who report medications for the CV risk factors of interest, determine whether those undertreated have lower medication adherence (i.e., lower mean MMAS-4 scores [t-test or Wilcoxon

rank-sum]) vs. those not undertreated. Finally, determine if different health-related behavioral attitudes ("worried", "collaborative" and "self-controlling")[69] are associated with differential rates of underdiagnosis and undertreatment [chi-square test]. *A priori,* we hypothesize that the proportion undertreated will be greatest among "self-controlling" survivors vs. "collaborative" and "worried" (least undertreated) survivors. In subanalyses, we will analyze underdiagnosed participants (i.e., newly diagnosed per 1st home visit) separately from those undertreated (i.e., existing diagnosis but not meeting standard therapeutic goals) relative to the referent group (those without any abnormal CV risk factor). We also will use a combined logistic regression model to determine if knowledge, medication adherence, and health-related behavioral attitudes remain associated with underdiagnosis and undertreatment (combined and separately) after multivariate adjustment. Exploratory covariates are similar as before, and include sex, current age, insurance status, healthcare utilization, self-perceived CV risk, and family history of CV disease.

Post-intervention analysis: Among Aim 2 participants (and their designated healthcare provider), we will examine the differences between intervention and control groups following the 2nd home visit with respect to 1) survivor knowledge [chi-square test], 2) survivor self-efficacy [t-test or Wilcoxon rank-sum], 3) survivor-reported medication adherence [t-test or Wilcoxon rank-sum], and 4) provider-reported knowledge and self-efficacy [chi-square tests] related to the care of childhood cancer survivors. All analyses will be intent-to-treat and include all survivors and providers with available end points. Subanalyses can stratify these outcomes by initial underdiagnosis vs. undertreatment status. *We also will analyze practice changes over the 1-year intervention period using clinical records.* This includes comparing [chi-square test] whether documentation rates differ across study arms of: 1) any CV-related screening, 2) CV-related interventions [both lifestyle and drug prescriptions], and 3) treatment intensity (**Table 7** earlier). If any survivors share the same healthcare provider, we will apply bootstrap methods to account for the effects of clustering. In secondary analyses, we will determine, via logistic regression adjusting for randomization status, whether providers of survivors who remain undertreated have lower self-reported knowledge and self-efficacy related to the care of childhood cancer survivors vs. providers of survivors no longer undertreated.

Sample size: For covariates assessed at baseline in Aim 3, we will be able to detect differences ≥5%, RRs ≤0.8 / ≥1.2, and ≥20% of 1 standard deviation (**Table 10**). Among randomized participants (240/arm) and subanalyses (e.g., analyzing undertreatment, underdiagnosis separately: ~75-150/arm), detectable differences will be less.

TABLE 10. *Minimum detectable differences (80% power; α=0.05) per chi-square (proportions, relative risks) or t-test (% standard deviation). * ~60% (480 of 800) with abnormal CV finding.*

Covariate frequency	Baseline*	Post-intervention & subanalyses		
	N=320:480	240:240	150:150	75:75
10% (e.g., primary care provider self-efficacy towards childhood cancer survivorship care[27,28])	5%/17%<br>RR 0.5/1.7	3%/20%<br>RR 0.3/2.0	2%/22%<br>RR 0.2/2.2	- /28%<br>RR -/2.8
25% (e.g., MHLC self-controlling or worried typologies[69]; survivor anthracycline self-knowledge[70])	17%/34%<br>RR 0.7/1.4	15%/37%<br>RR 0.6/1.5	12%/40%<br>RR 0.5/1.6	8%/47%<br>RR 0.3/1.9
60% (e.g., MHLC collaborating typology[69]; survivor radiotherapy self-knowledge[70]; high medication adherence[30,31,67,71,72])	50%/70%<br>RR 0.8/1.2	47%/72%<br>RR 0.8/1.2	44%/75%<br>RR 0.7/1.3	37%/81%<br>RR 0.6/1.3
% standard deviation (e.g., MMAS-4,[60] survivor self-efficacy[56])	20%	26%	33%	46%

<u>Potential pitfalls / solutions</u>: Self-reported medication adherence is not as accurate as data from pharmacy databases.[100] However, use of these databases is unlikely to be feasible even in large integrated health systems given the relative rarity of childhood cancer survivors. Given upfront randomization, any reporting biases should be similar across study arms. Should our study uncover a suggestive association with adherence, this would provide data to support the use of more expensive medication monitoring devices in a follow-up study.[100] We also anticipate some missing healthcare provider responses. It is possible that providers on the intervention arm who receive the SCP could be more motivated to respond (all providers will get clinically relevant results from home visits). We will closely monitor response rates for providers across the study arms, and will compare the characteristics of responding vs. non-responding providers using American Medical Association databases (physicians only) and publically available information from state licensing boards and the internet (physicians and other providers).[28,102] In sensitivity analyses, we can examine the differences across study arms after adjustment for characteristics that appear to differ between responding and non-responding primary healthcare providers.

13.4 Ethnic and Gender Distribution Chart

Projected Target Accrual
ETHNIC AND GENDER DISTRIBUTION CHART

TARGETED / PLANNED ENROLLMENT: Number of Subjects			
Ethnic Category	Sex / Gender		
	Females	Males	Total
Hispanic or Latino	54	34	88
Not Hispanic or Latino	428	284	712
Ethnic Category Total of All Subjects*			
Racial Categories			
American Indian / Alaska Native	1	0	1
Asian	11	11	22
Native Hawaiian or Other Pacific Islander	0	0	0
Black or African American	38	24	62
White	406	268	674
More Than One Race	26	15	41
Racial Categories: Total of All Subjects*	482	318	800

14.0 REFERENCES

1. Robison LL, Armstrong GT, Boice JD, et al: The Childhood Cancer Survivor Study: a National Cancer Institute-supported resource for outcome and intervention research. J Clin Oncol 27:2308-2318, 2009
2. Robison LL, Hudson MM: Survivors of childhood and adolescent cancer: life-long risks and responsibilities. Nat. Rev. Cancer 14:61-70, 2014
3. Oeffinger KC, Mertens AC, Sklar CA, et al: Chronic health conditions in adult survivors of childhood cancer. N. Engl J Med 355:1572-1582, 2006
4. Mertens AC, Liu Q, Neglia JP, et al: Cause-specific late mortality among 5-year survivors of childhood cancer: the Childhood Cancer Survivor Study. J Natl Cancer Inst 100:1368-1379, 2008
5. Hudson MM, Ness KK, Gurney JG, et al: Clinical ascertainment of health outcomes among adults treated for childhood cancer. JAMA 309:2371-2381, 2013
6. Lipshultz SE, Adams MJ, Colan SD, et al: Long-term cardiovascular toxicity in children, adolescents, and young adults who receive cancer therapy: pathophysiology, course, monitoring, management, prevention, and research directions: A scientific statement from the American Heart Association. Circulation 128:1927-1995, 2013
7. Armstrong GT, Oeffinger KC, Chen Y, et al: Modifiable risk factors and major cardiac events among adult survivors of childhood cancer. J. Clin. Oncol 31:3673-3680, 2013
8. Reulen RC, Winter DL, Frobisher C, et al: Long-term cause-specific mortality among survivors of childhood cancer. JAMA 304:172-179, 2010
9. Tukenova M, Guibout C, Oberlin O, et al: Role of cancer treatment in long-term overall and cardiovascular mortality after childhood cancer. J. Clin. Oncol 28:1308-1315, 2010
10. Kero AE, Jarvela LS, Arola M, et al: Cardiovascular morbidity in long-term survivors of early-onset cancer: a population-based study. Int. J Cancer 134:664-673, 2014
11. van der Pal HJ, van Dalen EC, Hauptmann M, et al: Cardiac function in 5-year survivors of childhood cancer: a long-term follow-up study. Arch. Intern. Med 170:1247-1255, 2010
12. Chow EJ, Chen Y, Kremer LC, et al: Individual prediction of heart failure among childhood cancer survivors. J Clin Oncol 33:394-402, 2015
13. Brouwer CA, Postma A, Hooimeijer HL, et al: Endothelial damage in long-term survivors of childhood cancer. J Clin Oncol 31:3906-3913, 2013
14. Baker KS, Chow EJ, Goodman PJ, et al: Impact of treatment exposures on cardiovascular risk and insulin resistance in childhood cancer survivors. Cancer Epidemiol. Biomarkers Prev 22:1954-1963, 2013
15. Chow EJ, Chen Y, Armstrong GT, et al: Individual risk prediction of major cardiovascular events after cancer: a Childhood Cancer Survivor Study report. J Clin Oncol 32, 2014
16. Armenian SH, Sun CL, Vase T, et al: Cardiovascular risk factors in hematopoietic cell transplantation survivors: role in development of subsequent cardiovascular disease. Blood 120:4505-4512, 2012
17. Chow EJ, Wong K, Lee SJ, et al: Late cardiovascular complications after hematopoietic cell transplantation. Biol. Blood Marrow Transplant 20:794-800, 2014
18. Dengel DR, Kelly AS, Zhang L, et al: Signs of early sub-clinical atherosclerosis in childhood cancer survivors. Pediatr. Blood Cancer 61:532-537, 2014
19. Oeffinger KC, Adams-Huet B, Victor RG, et al: Insulin resistance and risk factors for cardiovascular disease in young adult survivors of childhood acute lymphoblastic leukemia. J Clin Oncol 27:3698-3704, 2009

20. Chow EJ, Simmons JH, Roth CL, et al: Increased cardiometabolic traits in pediatric survivors of acute lymphoblastic leukemia treated with total body irradiation. Biol Blood Marrow Transplant 16:1674-1681, 2010
21. van Waas M, Neggers SJ, Pieters R, et al: Components of the metabolic syndrome in 500 adult long-term survivors of childhood cancer. Ann Oncol 21:1121-1126, 2010
22. Nathan PC, Daugherty CK, Wroblewski KE, et al: Family physician preferences and knowledge gaps regarding the care of adolescent and young adult survivors of childhood cancer. J Cancer Surviv 7:275-282, 2013
23. Suh E, Daugherty CK, Wroblewski K, et al: General internists' preferences and knowledge about the care of adult survivors of childhood cancer: a cross-sectional survey. Ann Intern Med 160:11-17, 2014
24. Shankar SM, Marina N, Hudson MM, et al: Monitoring for cardiovascular disease in survivors of childhood cancer: report from the Cardiovascular Disease Task Force of the Children's Oncology Group. Pediatrics 121:e387-e396, 2008
25. Nathan PC, Greenberg ML, Ness KK, et al: Medical care in long-term survivors of childhood cancer: a report from the childhood cancer survivor study. J Clin Oncol 26:4401-4409, 2008
26. Cox CL, Hudson MM, Mertens A, et al: Medical screening participation in the childhood cancer survivor study. Arch. Intern Med 169:454-462, 2009
27. Baldwin LM, Dobie SA, Cai Y, et al: Receipt of general medical care by colorectal cancer patients: a longitudinal study. J Am Board Fam. Med 24:57-68, 2011
28. Blaser BW, Kim HT, Alyea EP, III, et al: Hyperlipidemia and statin use after allogeneic hematopoietic stem cell transplantation. Biol Blood Marrow Transplant 18:575-583, 2012
29. Shin DW, Kim SY, Cho J, et al: Comparison of hypertension management between cancer survivors and the general public. Hypertens. Res 35:935-939, 2012
30. Calip GS, Boudreau DM, Loggers ET: Changes in adherence to statins and subsequent lipid profiles during and following breast cancer treatment. Breast Cancer Res. Treat 138:225-233, 2013
31. Calip GS, Hubbard RA, Stergachis A, et al: Adherence to oral diabetes medications and glycemic control during and following breast cancer treatment. Pharmacoepidemiol. Drug Saf 24:75-85, 2015
32. Cabana MD, Rand CS, Powe NR, et al: Why don't physicians follow clinical practice guidelines? A framework for improvement. JAMA 282:1458-1465, 1999
33. Khatib R, Schwalm JD, Yusuf S, et al: Patient and healthcare provider barriers to hypertension awareness, treatment and follow up: a systematic review and meta-analysis of qualitative and quantitative studies. PLoS. One 9:e84238, 2014
34. Committee on Cancer Survivorship: Improving Care and Quality of Life NCPB: From Cancer Patient to Cancer Survivor: Lost in Transition, in Hewitt M, Greenfield S, Stovall E (eds). Washington, D.C., Institute of Medicine and National Research Council, National Academies Press, 2006
35. Casillas J, Oeffinger KC, Hudson MM, et al: Identifying Predictors of Longitudinal Decline in the Level of Medical Care Received by Adult Survivors of Childhood Cancer: A Report from the Childhood Cancer Survivor Study. Health Serv. Res, 2015
36. Glasgow RE, Funnell MM, Bonomi AE, et al: Self-management aspects of the improving chronic illness care breakthrough series: implementation with diabetes and heart failure teams. Ann Behav. Med 24:80-87, 2002
37. Glasgow RE, Hampson SE, Strycker LA, et al: Personal-model beliefs and social-environmental barriers related to diabetes self-management. Diabetes Care 20:556-561, 1997
38. Lorig K, Ritter PL, Laurent DD, et al: Online diabetes self-management program: a randomized study. Diabetes Care 33:1275-1281, 2010
39. Schulman-Green D, Bradley EH, Knobf MT, et al: Self-management and transitions in women with advanced breast cancer. J Pain Symptom. Manage 42:517-525, 2011

40. Hudson MM, Neglia JP, Woods WG, et al: Lessons from the past: opportunities to improve childhood cancer survivor care through outcomes investigations of historical therapeutic approaches for pediatric hematological malignancies. Pediatr. Blood Cancer 58:334-343, 2012
41. Green DM, Kun LE, Matthay KK, et al: Relevance of historical therapeutic approaches to the contemporary treatment of pediatric solid tumors. Pediatr. Blood Cancer 60:1083-1094, 2013
42. Galea S, Tracy M: Participation rates in epidemiologic studies. Ann Epidemiol 17:643-653, 2007
43. Oeffinger KC, Hudson MM, Mertens AC, et al: Increasing rates of breast cancer and cardiac surveillance among high-risk survivors of childhood Hodgkin lymphoma following a mailed, one-page survivorship care plan. Pediatr. Blood Cancer 56:818-824, 2011
44. Syrjala KL, Stover AC, Yi JC, et al: Development and implementation of an Internet-based survivorship care program for cancer survivors treated with hematopoietic stem cell transplantation. J Cancer Surviv 5:292-304, 2011
45. Hudson MM, Leisenring W, Stratton KK, et al: Increasing cardiomyopathy screening in at-risk adult survivors of pediatric malignancies: a randomized controlled trial. J Clin Oncol 32:3974-3981, 2014
46. Hill-Kayser CE, Vachani CC, Hampshire MK, et al: Impact of internet-based cancer survivorship care plans on health care and lifestyle behaviors. Cancer 119:3854-3860, 2013
47. Chobanian AV, Bakris GL, Black HR, et al: The Seventh Report of the Joint National Committee on Prevention, Detection, Evaluation, and Treatment of High Blood Pressure: the JNC 7 report. JAMA 289:2560-72, 2003
48. James PA, Oparil S, Carter BL, et al: 2014 evidence-based guideline for the management of high blood pressure in adults: report from the panel members appointed to the Eighth Joint National Committee (JNC 8). JAMA 311:507-520, 2014
49. Eckel RH, Jakicic JM, Ard JD, et al: 2013 AHA/ACC guideline on lifestyle management to reduce cardiovascular risk: a report of the American College of Cardiology/American Heart Association Task Force on Practice Guidelines. Circulation 129:S76-99, 2014
50. Whelton PK, Carey RM, Aronow WS, et al: 2017 ACC/AHA/AAPA/ABC/ACPM/AGS/APhA/ASH/ASPC/NMA/PCNA Guideline for the Prevention, Detection, Evaluation, and Management of High Blood Pressure in Adults: A Report of the American College of Cardiology/American Heart Association Task Force on Clinical Practice Guidelines. Hypertension, 2017
51. Kavey RE, Allada V, Daniels SR, et al: Cardiovascular risk reduction in high-risk pediatric patients: a scientific statement from the American Heart Association Expert Panel on Population and Prevention Science; the Councils on Cardiovascular Disease in the Young, Epidemiology and Prevention, Nutrition, Physical Activity and Metabolism, High Blood Pressure Research, Cardiovascular Nursing, and the Kidney in Heart Disease; and the Interdisciplinary Working Group on Quality of Care and Outcomes Research: endorsed by the American Academy of Pediatrics. Circulation 114:2710-38, 2006
52. Expert panel on integrated guidelines for cardiovascular health and risk reduction in children and adolescents: summary report. Pediatrics 128 Suppl 5:S213-S256, 2011
53. Third Report of the National Cholesterol Education Program (NCEP) Expert Panel on Detection, Evaluation, and Treatment of High Blood Cholesterol in Adults (Adult Treatment Panel III) final report. Circulation 106:3143-3421, 2002
54. Standards of medical care in diabetes--2015: summary of revisions. Diabetes Care 38 Suppl:S4, 2015
55. Spain PD, Oeffinger KC, Candela J, et al: Response to a treatment summary and care plan among adult survivors of pediatric and young adult cancer. J Oncol Pract 8:196-202, 2012
56. Schwarzer R, Luszczynska A: Self-efficacy, 2015
57. Hinds PS, Nuss SL, Ruccione KS, et al: PROMIS pediatric measures in pediatric oncology: valid and clinically feasible indicators of patient-reported outcomes. Pediatr. Blood Cancer 60:402-408, 2013

58. Cella D, Choi S, Garcia S, et al: Setting standards for severity of common symptoms in oncology using the PROMIS item banks and expert judgment. Qual. Life Res, 2014
59. Wallston KA, Wallston BS, DeVellis R: Development of the Multidimensional Health Locus of Control (MHLC) Scales. Health Educ. Monogr 6:160-170, 1978
60. Morisky DE, Green LW, Levine DM: Concurrent and predictive validity of a self-reported measure of medication adherence. Med Care 24:67-74, 1986
61. Forsythe LP, Parry C, Alfano CM, et al: Use of survivorship care plans in the United States: associations with survivorship care. J Natl. Cancer Inst 105:1579-1587, 2013
62. Rosoff PM, Werner C, Clipp EC, et al: Response rates to a mailed survey targeting childhood cancer survivors: a comparison of conditional versus unconditional incentives. Cancer Epidemiol. Biomarkers Prev 14:1330-1332, 2005
63. Alexander GL, Divine GW, Couper MP, et al: Effect of incentives and mailing features on online health program enrollment. Am. J Prev. Med 34:382-388, 2008
64. Baker R, Camosso-Stefinovic J, Gillies C, et al: Tailored interventions to overcome identified barriers to change: effects on professional practice and health care outcomes. Cochrane. Database. Syst. Rev:CD005470, 2010
65. Unverzagt S, Peinemann F, Oemler M, et al: Meta-regression analyses to explain statistical heterogeneity in a systematic review of strategies for guideline implementation in primary health care. PLoS. One 9:e110619, 2014
66. Glynn LG, Murphy AW, Smith SM, et al: Interventions used to improve control of blood pressure in patients with hypertension. Cochrane. Database. Syst. Rev:CD005182, 2010
67. Schedlbauer A, Davies P, Fahey T: Interventions to improve adherence to lipid lowering medication. Cochrane. Database. Syst. Rev:CD004371, 2010
68. Wens J, Vermeire E, Hearnshaw H, et al: Educational interventions aiming at improving adherence to treatment recommendations in type 2 diabetes: A sub-analysis of a systematic review of randomised controlled trials. Diabetes Res. Clin Pract 79:377-388, 2008
69. Cox CL, Zhu L, Hudson MM, et al: Survivor typologies predict medical surveillance participation: the childhood cancer survivor study. Psychooncology 22:1534-1542, 2013
70. Kadan-Lottick NS, Robison LL, Gurney JG, et al: Childhood cancer survivors' knowledge about their past diagnosis and treatment: Childhood Cancer Survivor Study. JAMA 287:1832-1839, 2002
71. Schmittdiel JA, Uratsu CS, Karter AJ, et al: Why don't diabetes patients achieve recommended risk factor targets? Poor adherence versus lack of treatment intensification. J Gen. Intern Med 23:588-594, 2008
72. Daugherty SL, Powers JD, Magid DJ, et al: The association between medication adherence and treatment intensification with blood pressure control in resistant hypertension. Hypertension 60:303-309, 2012

15.0 APPENDICES

The following documents are available as appendix (supplemental) items:

- Approach (study introduction) letter
- Consent template
- Ineligible participant thank you letter
- Patient baseline questionnaire
- Patient follow-up questionnaire
- Healthcare provider cover letter
- Healthcare provider questionnaire
- Survivorship care plan template
- Action Plan template
- Counseling fidelity rating form
-

References

1. Robison LL, Armstrong GT, Boice JD, et al: The Childhood Cancer Survivor Study: a National Cancer Institute-supported resource for outcome and intervention research. J Clin Oncol 27:2308-2318, 2009
2. Robison LL, Hudson MM: Survivors of childhood and adolescent cancer: life-long risks and responsibilities. Nat. Rev. Cancer 14:61-70, 2014
3. Oeffinger KC, Mertens AC, Sklar CA, et al: Chronic health conditions in adult survivors of childhood cancer. N. Engl J Med 355:1572-1582, 2006
4. Mertens AC, Liu Q, Neglia JP, et al: Cause-specific late mortality among 5-year survivors of childhood cancer: the Childhood Cancer Survivor Study. J Natl Cancer Inst 100:1368-1379, 2008
5. Hudson MM, Ness KK, Gurney JG, et al: Clinical ascertainment of health outcomes among adults treated for childhood cancer. JAMA 309:2371-2381, 2013
6. Lipshultz SE, Adams MJ, Colan SD, et al: Long-term cardiovascular toxicity in children, adolescents, and young adults who receive cancer therapy: pathophysiology, course, monitoring, management, prevention, and research directions: a scientific statement from the American Heart Association. Circulation 128:1927-1995, 2013
7. Armstrong GT, Oeffinger KC, Chen Y, et al: Modifiable risk factors and major cardiac events among adult survivors of childhood cancer. J Clin Oncol 31:3673-80, 2013
8. Reulen RC, Winter DL, Frobisher C, et al: Long-term cause-specific mortality among survivors of childhood cancer. JAMA 304:172-179, 2010
9. Tukenova M, Guibout C, Oberlin O, et al: Role of cancer treatment in long-term overall and cardiovascular mortality after childhood cancer. J Clin Oncol 28:1308-1315, 2010
10. Kero AE, Jarvela LS, Arola M, et al: Cardiovascular morbidity in long-term survivors of early-onset cancer: a population-based study. Int. J Cancer 134:664-673, 2014
11. van der Pal HJ, van Dalen EC, Hauptmann M, et al: Cardiac function in 5-year survivors of childhood cancer: a long-term follow-up study. Arch. Intern. Med 170:1247-1255, 2010
12. Chow EJ, Chen Y, Kremer LC, et al: Individual prediction of heart failure among childhood cancer survivors. J Clin Oncol 33:394-402, 2015
13. Brouwer CA, Postma A, Hooimeijer HL, et al: Endothelial damage in long-term survivors of childhood cancer. J Clin Oncol 31:3906-3913, 2013
14. Baker KS, Chow EJ, Goodman PJ, et al: Impact of treatment exposures on cardiovascular risk and insulin resistance in childhood cancer survivors. Cancer Epidemiol. Biomarkers Prev 22:1954-1963, 2013

15. Chow EJ, Chen Y, Armstrong GT, et al: Individual risk prediction of major cardiovascular events after cancer: a Childhood Cancer Survivor Study report. J Clin Oncol 32, 2014
16. Armenian SH, Sun CL, Vase T, et al: Cardiovascular risk factors in hematopoietic cell transplantation survivors: role in development of subsequent cardiovascular disease. Blood 120:4505-4512, 2012
17. Chow EJ, Wong K, Lee SJ, et al: Late cardiovascular complications after hematopoietic cell transplantation. Biol. Blood Marrow Transplant 20:794-800, 2014
18. Dengel DR, Kelly AS, Zhang L, et al: Signs of early sub-clinical atherosclerosis in childhood cancer survivors. Pediatr. Blood Cancer 61:532-537, 2014
19. Oeffinger KC, Adams-Huet B, Victor RG, et al: Insulin resistance and risk factors for cardiovascular disease in young adult survivors of childhood acute lymphoblastic leukemia. J Clin Oncol 27:3698-3704, 2009
20. Chow EJ, Simmons JH, Roth CL, et al: Increased cardiometabolic traits in pediatric survivors of acute lymphoblastic leukemia treated with total body irradiation. Biol Blood Marrow Transplant 16:1674-1681, 2010
21. van Waas M, Neggers SJ, Pieters R, et al: Components of the metabolic syndrome in 500 adult long-term survivors of childhood cancer. Ann Oncol 21:1121-1126, 2010
22. Nathan PC, Daugherty CK, Wroblewski KE, et al: Family physician preferences and knowledge gaps regarding the care of adolescent and young adult survivors of childhood cancer. J Cancer Surviv 7:275-282, 2013
23. Suh E, Daugherty CK, Wroblewski K, et al: General internists' preferences and knowledge about the care of adult survivors of childhood cancer: a cross-sectional survey. Ann Intern Med 160:11-17, 2014
24. Shankar SM, Marina N, Hudson MM, et al: Monitoring for cardiovascular disease in survivors of childhood cancer: report from the Cardiovascular Disease Task Force of the Children's Oncology Group. Pediatrics 121:e387-e396, 2008
25. Nathan PC, Greenberg ML, Ness KK, et al: Medical care in long-term survivors of childhood cancer: a report from the childhood cancer survivor study. J Clin Oncol 26:4401-4409, 2008
26. Cox CL, Hudson MM, Mertens A, et al: Medical screening participation in the childhood cancer survivor study. Arch. Intern Med 169:454-462, 2009
27. Baldwin LM, Dobie SA, Cai Y, et al: Receipt of general medical care by colorectal cancer patients: a longitudinal study. J Am Board Fam. Med 24:57-68, 2011
28. Blaser BW, Kim HT, Alyea EP, III, et al: Hyperlipidemia and statin use after allogeneic hematopoietic stem cell transplantation. Biol Blood Marrow Transplant 18:575-583, 2012
29. Shin DW, Kim SY, Cho J, et al: Comparison of hypertension management between cancer survivors and the general public. Hypertens. Res 35:935-939, 2012
30. Calip GS, Boudreau DM, Loggers ET: Changes in adherence to statins and subsequent lipid profiles during and following breast cancer treatment. Breast Cancer Res. Treat 138:225-233, 2013
31. Calip GS, Hubbard RA, Stergachis A, et al: Adherence to oral diabetes medications and glycemic control during and following breast cancer treatment. Pharmacoepidemiol. Drug Saf 24:75-85, 2015
32. Cabana MD, Rand CS, Powe NR, et al: Why don't physicians follow clinical practice guidelines? A framework for improvement. JAMA 282:1458-1465, 1999
33. Khatib R, Schwalm JD, Yusuf S, et al: Patient and healthcare provider barriers to hypertension awareness, treatment and follow up: a systematic review and meta-analysis of qualitative and quantitative studies. PLoS. One 9:e84238, 2014
34. Committee on Cancer Survivorship: Improving Care and Quality of Life NCPB: From Cancer Patient to Cancer Survivor: Lost in Transition, in Hewitt M, Greenfield S, Stovall E (eds). Washington, D.C., Institute of Medicine and National Research Council, National Academies Press, 2006
35. Casillas J, Oeffinger KC, Hudson MM, et al: Identifying Predictors of Longitudinal Decline in the Level of Medical Care Received by Adult Survivors of Childhood Cancer: A Report from the Childhood Cancer Survivor Study. Health Serv. Res, 2015
36. Glasgow RE, Funnell MM, Bonomi AE, et al: Self-management aspects of the improving chronic illness care breakthrough series: implementation with diabetes and heart failure teams. Ann Behav. Med 24:80-87, 2002
37. Glasgow RE, Hampson SE, Strycker LA, et al: Personal-model beliefs and social-environmental barriers related to diabetes self-management. Diabetes Care 20:556-561, 1997

38. Lorig K, Ritter PL, Laurent DD, et al: Online diabetes self-management program: a randomized study. Diabetes Care 33:1275-1281, 2010

39. Schulman-Green D, Bradley EH, Knobf MT, et al: Self-management and transitions in women with advanced breast cancer. J Pain Symptom. Manage 42:517-525, 2011

40. Hudson MM, Neglia JP, Woods WG, et al: Lessons from the past: opportunities to improve childhood cancer survivor care through outcomes investigations of historical therapeutic approaches for pediatric hematological malignancies. Pediatr. Blood Cancer 58:334-343, 2012

41. Green DM, Kun LE, Matthay KK, et al: Relevance of historical therapeutic approaches to the contemporary treatment of pediatric solid tumors. Pediatr. Blood Cancer 60:1083-1094, 2013

42. Galea S, Tracy M: Participation rates in epidemiologic studies. Ann Epidemiol 17:643-653, 2007

43. Oeffinger KC, Hudson MM, Mertens AC, et al: Increasing rates of breast cancer and cardiac surveillance among high-risk survivors of childhood Hodgkin lymphoma following a mailed, one-page survivorship care plan. Pediatr. Blood Cancer 56:818-824, 2011

44. Syrjala KL, Stover AC, Yi JC, et al: Development and implementation of an Internet-based survivorship care program for cancer survivors treated with hematopoietic stem cell transplantation. J Cancer Surviv 5:292-304, 2011

45. Hudson MM, Leisenring W, Stratton KK, et al: Increasing cardiomyopathy screening in at-risk adult survivors of pediatric malignancies: a randomized controlled trial. J Clin Oncol 32:3974-3981, 2014

46. Hill-Kayser CE, Vachani CC, Hampshire MK, et al: Impact of internet-based cancer survivorship care plans on health care and lifestyle behaviors. Cancer 119:3854-3860, 2013

47. Chobanian AV, Bakris GL, Black HR, et al: The Seventh Report of the Joint National Committee on Prevention, Detection, Evaluation, and Treatment of High Blood Pressure: the JNC 7 report. JAMA 289:2560-72, 2003

48. James PA, Oparil S, Carter BL, et al: 2014 evidence-based guideline for the management of high blood pressure in adults: report from the panel members appointed to the Eighth Joint National Committee (JNC 8). JAMA 311:507-520, 2014

49. Eckel RH, Jakicic JM, Ard JD, et al: 2013 AHA/ACC guideline on lifestyle management to reduce cardiovascular risk: a report of the American College of Cardiology/American Heart Association Task Force on Practice Guidelines. Circulation 129:S76-99, 2014

50. Whelton PK, Carey RM, Aronow WS, et al: 2017 ACC/AHA/AAPA/ABC/ACPM/AGS/APhA/ASH/ASPC/NMA/PCNA Guideline for the Prevention, Detection, Evaluation, and Management of High Blood Pressure in Adults: A Report of the American College of Cardiology/American Heart Association Task Force on Clinical Practice Guidelines. Hypertension, 2017

51. Kavey RE, Allada V, Daniels SR, et al: Cardiovascular risk reduction in high-risk pediatric patients: a scientific statement from the American Heart Association Expert Panel on Population and Prevention Science; the Councils on Cardiovascular Disease in the Young, Epidemiology and Prevention, Nutrition, Physical Activity and Metabolism, High Blood Pressure Research, Cardiovascular Nursing, and the Kidney in Heart Disease; and the Interdisciplinary Working Group on Quality of Care and Outcomes Research: endorsed by the American Academy of Pediatrics. Circulation 114:2710-38, 2006

52. Expert panel on integrated guidelines for cardiovascular health and risk reduction in children and adolescents: summary report. Pediatrics 128 Suppl 5:S213-S256, 2011

53. Third Report of the National Cholesterol Education Program (NCEP) Expert Panel on Detection, Evaluation, and Treatment of High Blood Cholesterol in Adults (Adult Treatment Panel III) final report. Circulation 106:3143-3421, 2002

54. Standards of medical care in diabetes--2015: summary of revisions. Diabetes Care 38 Suppl:S4, 2015

55. Spain PD, Oeffinger KC, Candela J, et al: Response to a treatment summary and care plan among adult survivors of pediatric and young adult cancer. J Oncol Pract 8:196-202, 2012

56. Schwarzer R, Luszczynska A: Self-efficacy, 2015

57. Hinds PS, Nuss SL, Ruccione KS, et al: PROMIS pediatric measures in pediatric oncology: valid and clinically feasible indicators of patient-reported outcomes. Pediatr. Blood Cancer 60:402-408, 2013

58. Cella D, Choi S, Garcia S, et al: Setting standards for severity of common symptoms in oncology using the PROMIS item banks and expert judgment. Qual. Life Res, 2014

59. Wallston KA, Wallston BS, DeVellis R: Development of the Multidimensional Health Locus of Control (MHLC) Scales. Health Educ. Monogr 6:160-170, 1978
60. Morisky DE, Green LW, Levine DM: Concurrent and predictive validity of a self-reported measure of medication adherence. Med Care 24:67-74, 1986
61. Forsythe LP, Parry C, Alfano CM, et al: Use of survivorship care plans in the United States: associations with survivorship care. J Natl. Cancer Inst 105:1579-1587, 2013
62. Rosoff PM, Werner C, Clipp EC, et al: Response rates to a mailed survey targeting childhood cancer survivors: a comparison of conditional versus unconditional incentives. Cancer Epidemiol. Biomarkers Prev 14:1330-1332, 2005
63. Alexander GL, Divine GW, Couper MP, et al: Effect of incentives and mailing features on online health program enrollment. Am. J Prev. Med 34:382-388, 2008
64. Baker R, Camosso-Stefinovic J, Gillies C, et al: Tailored interventions to overcome identified barriers to change: effects on professional practice and health care outcomes. Cochrane. Database. Syst. Rev:CD005470, 2010
65. Unverzagt S, Peinemann F, Oemler M, et al: Meta-regression analyses to explain statistical heterogeneity in a systematic review of strategies for guideline implementation in primary health care. PLoS. One 9:e110619, 2014
66. Glynn LG, Murphy AW, Smith SM, et al: Interventions used to improve control of blood pressure in patients with hypertension. Cochrane. Database. Syst. Rev:CD005182, 2010
67. Schedlbauer A, Davies P, Fahey T: Interventions to improve adherence to lipid lowering medication. Cochrane. Database. Syst. Rev:CD004371, 2010
68. Wens J, Vermeire E, Hearnshaw H, et al: Educational interventions aiming at improving adherence to treatment recommendations in type 2 diabetes: A sub-analysis of a systematic review of randomised controlled trials. Diabetes Res. Clin Pract 79:377-388, 2008
69. Cox CL, Zhu L, Hudson MM, et al: Survivor typologies predict medical surveillance participation: the childhood cancer survivor study. Psychooncology 22:1534-1542, 2013
70. Kadan-Lottick NS, Robison LL, Gurney JG, et al: Childhood cancer survivors' knowledge about their past diagnosis and treatment: Childhood Cancer Survivor Study. JAMA 287:1832-1839, 2002
71. Schmittdiel JA, Uratsu CS, Karter AJ, et al: Why don't diabetes patients achieve recommended risk factor targets? Poor adherence versus lack of treatment intensification. J Gen. Intern Med 23:588-594, 2008
72. Daugherty SL, Powers JD, Magid DJ, et al: The association between medication adherence and treatment intensification with blood pressure control in resistant hypertension. Hypertension 60:303-309, 2012
