# Supplementary material for: Counseling and Cardiovascular Disease Risk Factor Control in Long-Term Cancer Survivors: A Randomized Clinical Trial
Source: JAMA Netw Open. 2026 Feb 5;9(2):e2555863. doi: 10.1001/jamanetworkopen.2025.55863 (PMC12878413; doi:10.1001/jamanetworkopen.2025.55863)
Supplement: Supplement 2. — eTable 1. US Metropolitan Areas Used for Study Recruitment eTable 2. Distribution of Undertreated Conditions Based on Home Visit Measurements by Randomization Status, Corresponding to Estimates Shown in Main Figure 1 eTable 3. Differences in Outcomes by Randomized Group, and Within Each Group at 1-Year Follow-Up vs Baseline eTable 4. Odds of Undertreatment Among Trial Participants After 1 Year in Relation to Change in Self-Reported Self-Efficacy and Baseline Multidimensional Health Locus of Control (MHLC) Domains eTable 5. Changes in Primary Health Care Provider Documentation After 1-Year Follow-Up by Randomization Status (293 of 347) eAppendix. Cancer Treatment Summary and Survivorship Care Plan [file jamanetwopen-e2555863-s002.pdf]

Supplementary Online Content

Chow EJ, Chen Y, Yasui Y, et al. Counseling and cardiovascular disease risk factor control in long-term cancer survivors: a randomized clinical trial. *JAMA Netw Open.* 2026;9(2):e2555863. doi:10.1001/jamanetworkopen.2025.55863

eTable 1. US Metropolitan Areas Used for Study Recruitment
eTable 2. Distribution of Undertreated Conditions Based on Home Visit Measurements by Randomization Status, Corresponding to Estimates Shown in Main Figure 1
eTable 3. Differences in Outcomes by Randomized Group, and Within Each Group at 1-Year Follow-Up vs Baseline
eTable 4. Odds of Undertreatment Among Trial Participants After 1 Year in Relation to Change in Self-Reported Self-Efficacy and Baseline Multidimensional Health Locus of Control (MHLC) Domains
eTable 5. Changes in Primary Health Care Provider Documentation After 1-Year Follow-Up by Randomization Status (293 of 347)
eAppendix. Cancer Treatment Summary and Survivorship Care Plan

This supplementary material has been provided by the authors to give readers additional information about their work.

© 2026 Chow EJ et al. *JAMA Network Open*.

eTable 1. US Metropolitan Areas Used for Study Recruitment

Atlanta, Georgia<br>Boston, Massachusetts<br>Columbus, Ohio<br>Denver, Colorado<br>Houston, Texas<br>Minneapolis, Minnesota<br>Philadelphia, Pennsylvania<br>Pittsburgh, Pennsylvania<br>Seattle, Washington	These 9 areas were chosen due to large concentrations of Childhood Cancer Survivor Study cohort members in the area and where the study's home examination company had field offices. Examiners were able to reach any participant within a 50-mile radius of their central field office.

eTable 2. Distribution of Undertreated Conditions Based on Home Visit Measurements by Randomization Status, Corresponding to Estimates Shown in Main Figure 1

Conditions, n (%)	Baseline		Follow-up		Intervention vs Control*	Follow-up vs baseline P-value	
	Intervention n=175	Control n=172	Intervention n=173	Control n=172	P-value	Intervention	Control
Hypertension	95 (54.3)	89 (51.7)	77 (44.5)	85 (49.4)	0.19	0.01	0.54
Dyslipidemia	95 (54.3)	85 (49.4)	92 (53.2)	67 (39.0)	0.009	0.72	0.004
Glucose intolerance	82 (46.9)	88 (51.2)	68 (39.3)	70 (40.7)	0.57	<0.001	<0.001
>1 condition	76 (43.4)	74 (43.0)	69 (39.9)	65 (37.8)	0.67	0.34	0.17
All 3 conditions	21 (12.0)	16 (9.3)	16 (9.3)	14 (8.1)	-	-	-

*Difference at follow-up, adjusting for the baseline status

© 2026 Chow EJ et al. *JAMA Network Open*.

eTable 3. Differences in Outcomes by Randomized Group, and Within Each Group at 1-Year Follow-Up vs Baseline

Outcomes	Difference between groups (95% CI)*	Group-specific difference at follow-up versus baseline (95% CI)†	
		Intervention	Enhanced care control
Systolic blood pressure, mmHg‡	-0.4 (-3.8, +3.0)	-4.6 (-7.1, -2.1)	-2.4 (-5.2, +0.3)
Diastolic blood pressure, mmHg‡	-0.7 (-3.1, +1.6)	-2.3 (-4.0, -0.5)	-1.9 (-3.7, -0.1)
Low density lipoprotein, mg/dL§	+1.5 (-8.9, +12.0)	-18.0 (-25.4, -10.6)	-20.1 (-28.9, -11.2)
Triglyceride, mg/dL§	+8.4 (-32.0, +48.8)	-8.5 (-39.8, +22.7)	-48.9 (-91.9, -5.8)
Blood glucose, mg/dL**	-10.7 (-22.7, +1.4)	-8.0 (-17.1, +1.2)	+5.5 (-3.8, +14.8)
Hemoglobin A1c, %**	-0.2 (-0.4, +0.1)	0.0 (-0.2, +0.2)	+0.2 (0.0, +0.3)

*Intervention versus control group (referent); adjusted for the baseline value
†Based on paired t-test
‡Among those randomized with undertreated hypertension at baseline, n=94 (intervention) and n=89 (control)
§Among those randomized with undertreated dyslipidemia at baseline, n=94 (intervention) and n=85 (control)
**Among those randomized with undertreated glucose intolerance at baseline, n=81 (intervention) and n=88 (control)

eTable 4. Odds of Undertreatment Among Trial Participants After 1 Year in Relation to Change in Self-Reported Self-Efficacy and Baseline Multidimensional Health Locus of Control (MHLC) Domains

Characteristic	OR (95% CI)*
Health-related self-efficacy†	
Lowest tertile	1.00 (ref)
Middle tertile	0.56 (0.31-1.01)
Highest tertile	0.64 (0.34-1.22)
MHLC domains‡	
Internal	1.02 (0.80-1.30)
Chance	0.93 (0.70-1.25)
Powerful others	1.42 (1.08-1.87)

*Adjusted for sex, current age, time since cancer diagnosis, history of survivorship clinic visit, obese body mass index, undertreated condition, and randomization assignment
†Assessed at baseline and at one-year follow-up
‡Only assessed at baseline, with estimates reflecting the association with a one-point change in each scale

© 2026 Chow EJ et al. *JAMA Network Open*.

eTable 5. Changes in Primary Health Care Provider Documentation After 1-Year Follow-Up by Randomization Status (293 of 347)

Documentation element	Baseline rates, %		Net improvement in documentation, %		P value†
	Intervention n=144	Control n=149	Intervention n=144	Control n=149	
Childhood cancer history	63.9	71.1	+18.5	+10.9	0.26
Radiation exposure	37.5	32.9	+12.5	+12.1	0.33
Cardiotoxic chemotherapy	8.3	10.1	+12.3	+1.9	0.03
Increased cardiovascular disease risk	14.6	10.1	+14.8	+0.9	0.002
Survivorship care plan	6.3	3.4	+20.2	-1.4	<0.001
Any late effects surveillance plan	34.7	30.2	+12.3	+6.2	0.06
Cardiac testing plan‡	29.9	28.2	-4.7	-5.7	0.59

*Limited to participants with available medical records
†Difference between the intervention versus control group, adjusting for each group's baseline value
‡Refers to electrocardiogram, echocardiogram, or other imaging being performed or planned

© 2026 Chow EJ et al. *JAMA Network Open*.

eAppendix. Cancer Treatment Summary and Survivorship Care Plan

**CANCER TREATMENT SUMMARY and SURVIVORSHIP CARE PLAN**	
**Name:**	**Study ID:**
**Date of Birth:**	**Age at Diagnosis:**
**Primary Healthcare Provider:**	
**Other Current Healthcare Provider(s):**	
**Cancer Treatment Summary***	
**Original Treatment Center:**	
**Cancer Diagnosis:**	**Disease Site:**
**Date of Diagnosis:**	**End of Therapy Date:**
**Bone Marrow Transplant:** [this row would only be listed if applicable]	
**Radiation:** [body area exposed, dose if known]	
**Cumulative Anthracycline Dose (if known):** [list cumulative dose for each agent]<br><br>**Cumulative Alkylating Agent Dose (if known):** [list cumulative dose for each agent]<br><br>**Other Chemotherapy:** [each agent listed]	

**This information was taken from medical records available for your original cancer diagnosis from your original pediatric treatment center as part of your participation in the Long Term Follow-Up Study. If you feel any of this information may be incorrect, please let us know. Information on any second/subsequent cancers you may have since experienced is not included in this summary.*

Any questions from you or your primary healthcare provider related to this document can be directed to:

Eric Chow, MD MPH
Fred Hutchinson Cancer Research Center

Phone: 1-866-325-9525
Email: chiipstudy@fredhutch.org

Prepared for: ______________________; Date Prepared: ______

© 2026 Chow EJ et al. *JAMA Network Open*.

Potential Health Problems Related to Your Cancer Treatment

based on the Children's Oncology Group Long-term Follow-Up Guidelines: www.survivorshipguidelines.org

[Template only – not all organ systems will be listed as it will depend on prior treatment exposures in <YELLOW> highlight]

Heart. Anthracyclines (doxorubicin, daunorubicin, epirubicin, idarubicin, mitoxantrone [keep only those that were given]) and radiation (including scatter from abdominal, spinal radiation) [keep language re: scatter if relevant, keep only those fields that are applicable] may cause heart problems. [If ≥40Gy neck radiation] This can include early carotid (neck) artery disease.

Lungs. Radiation, bleomycin, CCNU (lomustine), BCNU (carmustine), busulfan may cause scarring of the lungs.

Learning/Brain function. Intrathecal chemotherapy (directly injected into the spinal fluid), cranial radiation may cause some learning issues, including memory problems.

Eyes. Radiation, steroids may cause cataracts.

Hearing. Cisplatin, carboplatin, radiation [if ≥30Gy] may cause hearing problems.

Dental. Chemotherapy, radiation can cause dental abnormalities. Recommend dental evaluations every 6 months.

Endocrine/hormones. Radiation, alkylators (cyclophosphamide, ifosfamide, busulfan, lomustine, melphalan, procarbazine, thiotepa [keep only those that were given]) can cause a variety of hormone problems. In your case, this may include effects on [select relevant] thyroid [any radiation to brain/neck], pituitary (in the brain) [if cranial RT ≥30Gy], ovaries/testes [any radiation to those areas; also if any alkylator chemotherapy]. Radiation can increase the likelihood of having abnormal cholesterol/triglyceride levels [if total body radiation] and diabetes [any abdominal/flank radiation].

Kidney/urinary. Cisplatin, carboplatin, ifosfamide, radiation, bone marrow transplant can damage the kidneys. Radiation [if ≥30Gy to bladder] can cause bladder problems.

Gastrointestinal. Surgery, abdominal radiation [if ≥30Gy] can lead to stomach and intestinal symptoms.

Musculoskeletal. Radiation can cause reduced or uneven growth. Steroids can lead to low bone mineral density, which may influence your risk of future bone fractures.

Skin. Radiation increases your risk for skin cancers. Please use appropriate sun protection, avoid sun burns and tanning booths.

Psychosocial. Survivors and their families could possibly experience emotional problems and/or difficulty or delay in returning to normal home and school life. This could occur even many years after treatment.

Cancer Prevention. Chest radiation [if ≥10 Gy] increases your risk of breast cancer. Abdominal radiation [if ≥30 Gy] increases your risk of colorectal cancer.

Prepared for: ______________________; Date Prepared: ______

© 2026 Chow EJ et al. *JAMA Network Open*.

**Results of Home Visit Testing**		
**Date of testing:**		**Hours fasted:**
**Test**	**Result**	**Comment** ***[only section corresponding to result will be shown]***
**Blood pressure**<br>(average of 2 measurements)		[NORMAL. Pressures <120 / <80 mmHg are ideal. Pressures 120-129 / <80 mmHg maybe concerning for high blood pressure in the future.]<br>[HIGH. Pressures 130 / 80 mmHg and greater are concerning for hypertension. Diagnosis of hypertension requires repeat testing. We strongly encourage follow-up with your healthcare provider.<br>[<<if 130+/80+ & patient has known hypertension>> HIGH Pressures <120 / <80 mmHg are normal. Please review what your goal blood pressures should be with your healthcare provider.]<br>[<<if 120-129 / <80 mmHg & patient has known hypertension>> Please review what your goal blood pressures should be with your healthcare provider.]
**LDL Cholesterol**<br>(LDL, low-density lipoprotein)		[NORMAL]<br>[HIGH. The American Heart Association and American Academy of Pediatrics consider LDL cholesterol 160 mg/dL and greater to be high for survivors of childhood cancer. We encourage follow-up with your healthcare provider.]<br>[<<if LDL not able to be calculated>> LDL calculation not valid for samples with triglyceride greater than 400 mg/dL. Total cholesterol measured was XXX mg/dL, HDL was XX mg/dL]
**Triglyceride**		[NORMAL for non-fasting sample (<10 hours) / fasting sample]<br>[HIGH. The American Heart Association and American Academy of Pediatrics consider triglyceride 150 mg/dL and greater to be high for survivors of childhood cancer. In non-fasting conditions (<10 hours fasted), levels 200 mg/dL and greater may be high. We encourage follow-up with your healthcare provider.]
**Glucose**<br>(blood sugar)		[NORMAL for non-fasting sample (<8 hours) / fasting sample]<br>[BORDERLINE. Fasting blood glucose 100-125 mg/dL is concerning for diabetes in the future. In non-fasting conditions (<8 hours fasted), a level 140-199 mg/dL is concerning for diabetes in the future. We encourage you to follow-up with your healthcare provider.]<br>[HIGH. Fasting blood glucose 126 mg/dL and greater is concerning for diabetes. In non-fasting conditions (<8 hours fasted), a level 200 mg/dL and greater is concerning for diabetes in the future. Diagnosis usually requires repeat testing. We strongly encourage follow-up with your healthcare provider.]<br>[<<if patient is a known diabetic>> Please review what your goal blood sugars and Hemoglobin A1Cs should be with your healthcare provider.]
**Hemoglobin A1C**<br>(another way of measuring blood sugar)		[NORMAL]<br>[BORDERLINE. Hemoglobin A1C 5.7-6.4% is concerning for diabetes in the future. We encourage follow-up with your healthcare provider.]

Prepared for: ______________________; Date Prepared: ______

© 2026 Chow EJ et al. *JAMA Network Open*.

	[HIGH. Hemoglobin A1C 6.5% and greater is concerning for diabetes. Diagnosis usually requires repeat testing. We strongly encourage follow-up with your healthcare provider.]<br>[<<If patient is a known diabetic & HbA1c ≥7.0%>> Hemoglobin A1C 7.0% or greater may show less than ideal blood sugar control. Please review what your goal Hemoglobin A1C should be with your healthcare provider.]<br>[<<If patient is a known diabetic & HbA1c <7.0%>> Please review what your goal Hemoglobin A1C should be with your healthcare provider.]
We also measured your height, weight, and body mass index (BMI) during the visit:	
**Height:**<br>**Weight:**<br>**Body mass index:**	BMI is a measure of body fat based on your height and weight. BMI less than 18.5 is concerning for being underweight. 18.5 to 24.9 is considered the normal range. 25 to 29.9 is concerning for being overweight. 30 and greater is concerning for obesity. *For more information:* www.cdc.gov/healthyweight/

**Summary Recommendations for Follow-up Care *[Example only]***

Based on the cancer treatment you received as a child, you may be at increased risk for **early heart disease**.

- 7 to 11% chance of developing heart failure by age 50, with risk further increasing with age. This is comparable to a 7 to 20-fold increased risk compared with other people your age who did not have cancer as children. ***[Moderate HF risk group]***
- 9 to 16% chance of developing heart failure by age 50, with risk further increasing with age. This is comparable to a 20 to 75-fold increased risk compared with other people your age who did not have cancer as children. ***[High HF risk group]***
- 9 to 15% chance of developing coronary artery disease by age 50, with risk further increasing with age. This is comparable to a 5 to 10-fold increased risk compared with other people your age who did not have cancer as children. ***[Moderate CAD risk group]***
- 15 to 25% chance of developing coronary artery disease by age 50, with risk further increasing with age. This is comparable to a 10 to 30-fold increased risk compared with other people your age who did not have cancer as children. ***[High CAD risk group]***
- Other conditions such as high blood pressure (hypertension), abnormal cholesterol/triglyceride (lipid) levels, and high blood sugar (diabetes) can further increase your risk of early heart disease. Your home visit results today show that you may have:
  - Borderline high blood pressure / High blood pressure, [if previously NOT diagnosed, then also insert appropriate following language:] concerning for hypertension in the future [if systolic 120-129] / hypertension [if systolic 130+ or diastolic 80+]
  - Abnormal cholesterol/triglyceride levels
  - Borderline high blood sugar / High blood sugar, [based on either abnormal glucose OR HbA1c (if known diabetic, base this statement as HbA1c value, and leave as "High blood sugar" if HbA1c ≥7%); if previously NOT diagnosed, then also insert appropriate following language:] concerning for diabetes in the future [if glucose or HbA1c in borderline range] / diabetes [if glucose or HbA1c in high range]
- ***We strongly recommend you discuss these results with your primary healthcare provider.***

Prepared for: ______________________; Date Prepared: ______

© 2026 Chow EJ et al. *JAMA Network Open*.

- In addition, the Children's Oncology Group guidelines recommend a baseline electrocardiogram (ECG/EKG) if this has never been done, and periodic check-ups of heart function using an echocardiogram (ultrasound of the heart) or alternative screening [if exposed to heart radiation or anthracycline]. A physical exam of neck blood vessels, with or without an ultrasound, may also be important [if ≥40Gy neck radiation]. You should discuss with your primary healthcare provider whether any of these tests are necessary.
- *Finally, keep healthy habits,* such as regular exercise (adults are recommended to get 150 minutes each week of moderate-intensity activity like brisk walking or 75 minutes each week of vigorous activity like jogging or swimming), a well-balanced diet, and avoid smoking and tobacco products, and drinking too much alcohol.

[This section, only if relevant past exposures present] **Regarding new cancers,** given your past cancer treatment, current Children's Oncology Group guidelines specifically recommend:

- Screening for breast cancer for those who received 10 Gy or greater radiation dose to the breasts: clinical breast exam every 6 to 12 months, and beginning 8 years after radiation or age 25, whichever is later, consider annual mammogram and breast MRI.
- Screening for colorectal cancer for those who received 30 Gy or greater radiation dose to the abdomen: beginning 10 years after radiation or age 35, whichever is later.

A copy of this document including all home visit results will be mailed to your primary healthcare provider as well. Your provider may decide to repeat some of the testing.

Where Can I Find More Information? *[For Participants Only]*

Information specifically for survivors of childhood cancer

- Children's Oncology Group "Health Links" on various health topics relevant to survivors of childhood cancer: www.survivorshipguidelines.org
- Children's Oncology Group also maintains a nationwide directory of clinics that provide dedicated care for survivors of childhood cancer: http://applications.childrensoncologygroup.org/Surveys/lateEffects/lateEffects.PublicSearch.asp
- National Cancer Institute also offers information on health issues relevant to cancer survivors: http://www.cancer.gov/types/childhood-cancers/late-effects-pdq

More general information on heart disease, heart health, and diabetes

- US Centers for Disease Control and Prevention:
  - General information: https://www.cdc.gov/heartdisease/
  - To quit smoking: https://www.cdc.gov/tobacco/quit_smoking/how_to_quit/resources/index.htm
- American Heart Association's tips for healthy heart lifestyles: https://healthyforgood.heart.org/
- American Diabetes Association: http://www.diabetes.org/

© 2026 Chow EJ et al. *JAMA Network Open*.

Information Resources for Healthcare Providers

Information designed specifically for survivors of childhood cancer

- Children's Oncology Group Long-Term Follow-Up guidelines has detailed information on health risks and recommended screening for survivors of childhood cancer: www.survivorshipguidelines.org
- Children's Oncology Group also maintains a nationwide directory of clinics that provide dedicated care for survivors of childhood cancer: http://applications.childrensoncologygroup.org/Surveys/lateEffects/lateEffects.PublicSearch.asp
- National Cancer Institute also offers detailed information on health issues relevant to cancer survivors: http://www.cancer.gov/types/childhood-cancers/late-effects-hp-pdq

Cardiovascular disease risk calculators

- General population 10-year risk for heart attack: http://cvdrisk.nhlbi.nih.gov/
- Childhood cancer survivor cardiovascular disease risk calculator (based on prior cancer treatment history): https://ccss.stjude.org/cvcalc

Other references

- Eckel RH, Jakicic JM, Ard JD, et al. 2013 AHA/ACC guideline on lifestyle management to reduce cardiovascular risk: a report of the American College of Cardiology/American Heart Association Task Force on Practice Guidelines. *Circulation* 2014;129(25 Suppl 2):S76-99.
- Expert panel on integrated guidelines for cardiovascular health and risk reduction in children and adolescents: summary report. *Pediatrics* 2011;128;S213.
- Kavey RE, Allada V, Daniels SR, et al. Cardiovascular risk reduction in high-risk pediatric patients: a scientific statement from the American Heart Association Expert Panel on Population and Prevention Science; the Councils on Cardiovascular Disease in the Young, Epidemiology and Prevention, Nutrition, Physical Activity and Metabolism, High Blood Pressure Research, Cardiovascular Nursing, and the Kidney in Heart Disease; and the Interdisciplinary Working Group on Quality of Care and Outcomes Research: endorsed by the American Academy of Pediatrics. *Circulation* 2006;114:2710-38.
- Lipshultz SE, Adams J, Colan SD, et al. Long-term cardiovascular toxicity in children, adolescents, and young adults who receive cancer therapy: pathophysiology, course, monitoring, management, prevention, and research directions: a scientific statemetn from the American Heart Association. *Circulation* 2013;128:1927-95.
- Standards of medical care in diabetes 2017: summary of revisions. *Diabetes Care* 2017;40(Suppl 1).
- Stone NJ, Robinson JG, Lichtenstein AH, et al. Treatment of blood cholesterol to reduce atherosclerotic cardiovascular disease risk in adults: synopsis of the 2013 ACC/AHA cholesterol guideline. *Ann Intern Med* 2014;160:339-43.
- Whelton PK, Carey RM, Aronow WS, et al. 2017 ACC… PCNA guideline for the prevention, detection, evaluation, and management of high blood pressure in adults: a report of the American College of Cardiology/American Heart Association Task Force on Clinical Practice Guidelines. *J Am Coll Cardiol* 2017 Nov 7. pii: S0735-1097(17)41519-1.

Prepared for: ______________________; Date Prepared: ______

© 2026 Chow EJ et al. *JAMA Network Open*.
