# Supplementary material for: Counseling and Cardiovascular Disease Risk Factor Control in Long-Term Cancer Survivors: A Randomized Clinical Trial
Source: JAMA Netw Open. 2026 Feb 5;9(2):e2555863. doi: 10.1001/jamanetworkopen.2025.55863 (PMC12878413; doi:10.1001/jamanetworkopen.2025.55863)
Supplement: Supplement 3. — Data Sharing Statement [file jamanetwopen-e2555863-s003.pdf]

# Data Sharing Statement

Chow. Counseling and Cardiovascular Disease Risk Factor Control in Long-Term Cancer Survivors. *JAMA Netw Open*. Published February 05, 2026.
doi:10.1001/jamanetworkopen.2025.55863

**Data**
**Additional Information:** Clinicaltrials.gov: NCT03104543
**Data available:** Yes
**Data types:** Deidentified participant data, Participant data with identifiers, Data dictionary
**How to access data:** http://ccss.stjude.org, https://survivorship.stjude.cloud/
**When available:** With publication

**Supporting Documents**
**Document types:** None

**Additional Information**
**Who can access the data:** There is a process on the CCSS website for researchers who request access to participant data with identifiers. This includes formal review of the proposal. There is separately an open access option without identifiers that is available through the St. Jude Cloud.
**Types of analyses:** Type of access depends on amoount of review and collaboration a researcher is interested in. We spell out the options on the data sharing statement on the cover page of the manuscript.
**Mechanisms of data availability:** The more formal process of working with CCSS comes with CCSS' statistical support. The completely open process of simply downloading deidentified data does not come with any research support. No data access agreement is required.
**Any additional restrictions:** Data users of deidentified data are not allowed to attempt to re-identify participants.
